# US grass-fed beef is as carbon intensive as industrial beef and ≈10-fold more intensive than common protein-dense alternatives

**DOI:** 10.1073/pnas.2404329122

**Published:** 2025-03-17

**Authors:** Gidon Eshel, Avi I. Flamholz, Alon Alon Shepon, Ron Milo

**Affiliations:** ^a^Department of Environmental Science, Bard College, Annandale-on-Hudson, NY 12504-5000; ^b^Laboratory of Environmental Microbiology, The Rockefeller University, New York, NY 10065; ^c^The Steinhardt Museum of Natural History, Tel Aviv University, Tel Aviv 61390, Israel; ^d^Department of Plant and Environmental Science, Weizmann Inst., Rehovot 7610001, Israel

**Keywords:** agricultural efficiency, beef, grazing, rangeland, carbon balance

## Abstract

The high resource intensity of industrial beef in high-income economies has prompted growing interest in alternative, potentially lower environmental impact beef production pathways. Of those, grass feeding is promoted by some as one such alternative, but rigorous quantification of this claim is required. Motivated to bridge this knowledge gap, we integrate empirical evidence with a model based on authoritative equations governing beef cattle performance to quantify the greenhouse gas emissions of producing grass-fed beef. Because geographical specificity and dependence on agricultural intensity are key, we model widely varied herds, from extensive operations on semiarid, marginal rangelands to partially industrial, intensive ones in lusher, more accommodating settings. We find that emissions per kg protein of even the most efficient grass-fed beef are 10 to 25% higher than those of industrial US beef and 3- to over 40-fold higher than a wide range of plant and animal alternatives. Soil sequestration enhancement by rangeland grazing reduces these emissions from 280–390 to 180–290 kg CO_2eq_ (kg protein)^−1^, still somewhat above industrial beef’s 180 to 220 kg CO_2eq_ (kg protein)^−1^, and well above nonbeef alternatives’ 10 to 70 kg CO_2eq_ (kg protein)^−1^. These differences prove robust across a broad set of combinations of grass-fed beef operation types, management practices, and ration qualities. Consequently, even with maximal credit for putative sequestration enhancement, grass-fed beef is still no less carbon intensive than industrial beef, and severalfold more intensive than nonbeef alternatives.

## Significance

Beef is more resource intensive per gram of edible protein than most other food items. Yet, grass-fed beef is sometimes promoted as environmentally desirable based on the expectation that cattle grazing may enhance soil carbon sequestration, thus offsetting production emissions. We quantitatively examine this view by integrating empirical observations with a beef herd model that uses standard animal science equations. We find that even under optimistic rangeland sequestration, grass-fed beef is not less carbon intensive than industrial beef and 3 to 40 times as carbon intensive as most plant and animal alternatives.

Averaged over all production systems, beef is the most resource intensive of all common foods (1, 2). In the United States, e.g., beef production requires, on average, 10 to 50 times as much land, irrigation, and fertilizer per kg protein as poultry or pork (3–7) while emitting >10 times more greenhouse gas. Consequently, while beef contributes only ≈5 to 20% of the calories and protein in a US diet, its production dominates food production related resource use in the United States (3, 8) and globally (9), undermining diet sustainability (8–17). This dominance is unambiguously evident in data and is faithfully simulated using various modeling techniques, life cycle assessments in particular, on global (e.g., UN-FAO GLEAM model), national (18–20), regional (19), and farm (21) scales.

This evidence has two potential caveats. First, while it characterizes industrial beef, it may mischaracterize grass-fed beef which can use lands unfit for human food production other than ruminant meat, pithily “cattle eat what humans cannot.” Second, grazing may promote soil carbon sequestration (22–24), mitigating some of the high greenhouse gas emissions of beef (25–27). While intriguing (22, 28), current estimates of U.S. beef production emissions do not adequately resolve this possibility, because by focusing on industrial feedlots (29), they mostly disregard such alternative production pathways as dairy beef (30), or mixed cropping and grass-fed beef (31, 32). What is more, many analyses have found the carbon intensity of industrial beef to be lower than that of grass-fed beef (29, 31–34). Some have also highlighted potential benefits of grazing to biodiversity (35, 36), surface hydrology (37), nutrient cycling (38), among other potential benefits.

The view of grass-fed beef as environmentally beneficial is intuitively appealing—Earth’s grasslands naturally yield bountiful forage, and grazing cattle appear to consume only this biomass and industrial refuse (“byproducts”), conforming with the cattle eat what humans cannot notion—but are not robust observations. Most importantly, it is not clear that cattle grazing indeed enhances average soil carbon sequestration (39), and—even if it does—that this is impactful enough to reverse beef’s carbon intensity (40, 41). The reason is that grazing raises required emissions per kg weight gain. For one thing, the elevated cellulose and lignin content of grazed forage (42–44) increases methane emissions per kg protein produced. In addition, grazing is seasonal (45) in most geographies, which requires fossil fuel-based, CO_2_-intensive supplemental off-season feed—grain, hay, or silage—that elevates production emissions. Beyond emissions, these feeds also compete with human food production for high-quality cropland and often also for irrigation water and agrochemicals (46).

A key challenge in this ongoing (34, 47–49) and unresolved (50, 51) discussion arises from the fact that sequestration rates are uncertain, likely of order ±10 to 100 kg C ha^−1^ y^−1^ on average [(52–54); ha = hectare = 10^4^ m^2^ ] but widely varied in magnitude and sign, with some localized observations suggesting values as high as $\pm 10^{3}$ kg C ha^−1^ y^−1^ (27, 55, 56). This is further complicated by the fact that ungrazed grasslands also sequester carbon, often more than grazed ones (40, 57, 58). In calculating carbon competitiveness, therefore, emissions incurred during beef production must be reduced not by total sequestration, but only by the *added* sequestration directly attributable to cattle grazing, which is roughly equally likely (59, 60) to be positive or negative (40, 57, 58). Net emissions (sequestration corrected; production emissions minus sequestration) thus fall below raw production emissions where cattle grazing enhances sequestration but exceed production emissions where cattle undermine sequestration (40, 57, 58). The nationwide effect, which has yet to be determined, would reflect the area weighted mean added sequestration, taking note of areas in which grazing enhances, or undermines, sequestration.

Here, we aim to better quantify the sequestration-corrected carbon costs or benefits of grass-fed beef and compare them to existing alternatives under widely varying geographies, agricultural practices, and sequestration rates. To do so rigorously, we combine standard equations (43) of beef cattle performance into a model, and use it to simulate overall feed needs and methane emissions of standard beef herds (*Methods*). From these emissions [recast as CO_2eq_ using the IPCC GWP100 conversion factor (61)], and literature-based values of the fraction of total CO_2eq_ emissions to which methane accounts, we then estimate total grass-fed beef CO_2eq_ emissions (*Methods*) and compare them to those of industrial beef and of non-beef alternatives.

We focus on the United States, primarily because of its relatively robust agricultural and carbon sequestration data coverage (62–64) and its global prominence in beef production and consumption (19) and in shaping dietary–cultural preferences (65). Nonetheless, as this focus only directly impacts chosen ranges of model parameters (*Methods*), and US beef ranching employs many practices that are used in other countries, our findings likely apply broadly to developed economies. The findings and discussion do not, however, apply to developing economies [such as those of, e.g., sub Saharan Africa or India (25). This limitation arises from the distinction between subsistence herding, typical of low-income economies, and industrial beef, which dominates the United States and other high-income nations. Industrial beef comprises two distinct, mostly geographically nonoverlapping, operations. In the first phase, termed cow-calf operations, which relies mostly on grass from a mixture of range and marginal cropland plus supplemental processed feed in winter, stock dams yield weaned offspring. While some weaned offspring replace culled bulls and dams, most enter the second, “finishing” operation, where they rapidly gain the bulk of their final weight by consuming very energy-dense, roughage poor rations derived almost exclusively from fine croplands.

The range of sequestration rates considered in our analysis reflects true rangelands (i.e., low productivity semiarid lands) or slightly lusher, more productive grasslands. It does not reflect pastures and meadows occupying croplands (or potential croplands), where the largest added sequestration rates, which can suppress production emissions to competitively low levels, are mostly observed (27, 51, 55, 56). Consequently, the settings most likely to render grass-fed beef carbon competitive are not considered. This is motivated by the fact that while grass-fed beef that graze productive croplands may appear more carbon efficient than our results indicate, this is misleading because such fine croplands can be repurposed from beef grazing (66) to producing most nonbeef foods (31, 56, 67). When such croplands are reallocated from beef production to the production of alternative food for direct human consumption, they deliver 3 to 7 times more protein—including all essential amino acids (63, 68)—per kg CO_2eq_ emitted, hectare occupied, or kg reactive nitrogen used (69), and supply 10-fold more of most known protective micronutrients per unit resource used (69), promoting public health (3–5, 7, 8, 70). This is why we omit the high sequestration rates that are sometimes observed on such lush agricultural croplands, focusing expressly on precipitation limited marginal lands in wealthy nations, lands that can only yield human food via grazing-based ruminant meat that does not undercut production of food for direct human consumption. Consistently, the primary question we pose is “Are net (sequestration corrected) emissions of beef grown on true rangelands, conforming with the cattle eat what humans cannot adage, indeed lower than those of industrial beef?”

Adequately resolving the distinct carbon calculus (71) of arid, semiarid, or minimally lush grasslands requires faithfully representing the coupled effects of precipitation, productivity, and agricultural intensity (72). Unfortunately, neither is an explicit input into the standard equations of beef cattle nutrition (*Methods*). We overcome this hurdle by using a proxy that loosely tracks the above covariates, namely ration metabolizable energy (ME) density in Mcal (10^3^ kcal) per kg dry feed, as described and justified in the *Methods*. In the presented analysis, therefore, we represent rangeland-based extensive low-productivity operations by 1.8 ≲ME≲2 Mcal (kg dry feed)^−1^, and semi-intensive operations using high productivity grasslands by 2.5≲ME≲3 Mcal (kg dry feed)^−1^.

## Results

### Total Operational Emissions of Grass-Fed Beef.

**Fig. 1.**
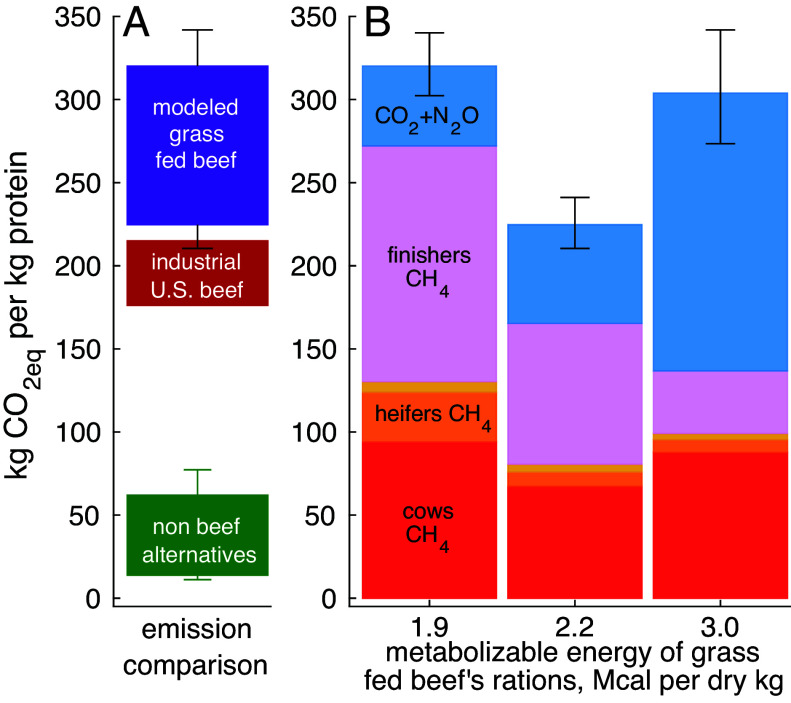
Operational emissions required for producing a kg of protein. In (*A*) the overall range spanned by all simulated grass-fed beef herds is shown in purple, comparing favorably with estimates for northeastern US grass-fed beef, 300 kg CO_2eq_ (kg protein)^−1^ (30). This range can be compared with emissions of US beef (mostly industrial; maroon red). The rectangle top, 220 kg CO_2eq_ (kg protein)^−1^, corresponds to a grass-fed beef herd that uses mostly fine Midwestern cropland (31). Its bottom reports the mean US national beef enterprise, mostly industrial, 180 kg CO_2eq_ (kg protein)^−1^ (19). Also in a, green shading shows emission statistics of 1,000 random combinations of 5 nonbeef alternative items (*SI Appendix*, section S3). Filled rectangles and whiskers report 10th to 90th and 5th to 95th percentile ranges, respectively. In (*B*) grass-fed beef emissions from a (purple) are split by ration quality (quantified as ME, Mcal per kg dry rations, horizontal axis), which acts as a proxy for agricultural intensity, as described in the text. The 4 filled colored bar sections report methane-related CO_2eq_ emission due to (from the *Bottom* upward) cows, heifers (first pregnancy females raised to replace culled cows), bulls plus their replacement young males, and finisher steers, the herd’s main beef source. See *Methods* for further detail.

**Fig. 2.**
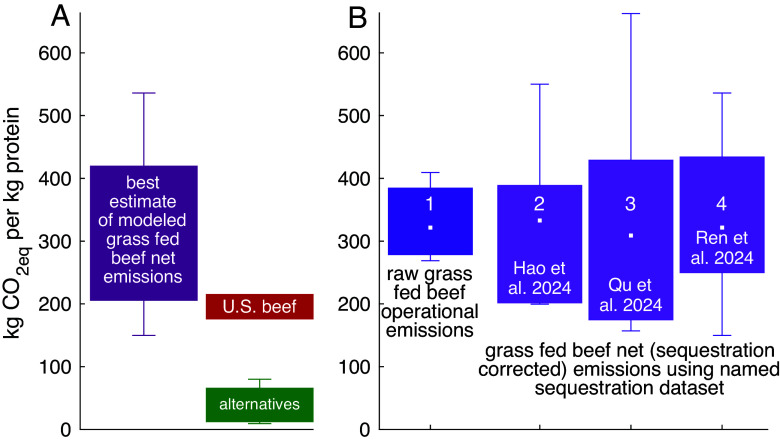
Emissions per kg protein in four model-simulated grass-fed beef operations compared to industrial beef and nonbeef alternatives. (*A*) Even after correcting operational emissions (*B*1) for reasonably expected soil sequestration (*B* 2 to 4), most grass-fed beef is still more carbon intensive than U.S. industrial beef (a, red), and far more than diverse protein conserving 5 nonbeef alternatives (a, green; *SI Appendix*, section S3). (*A*) “Modeled grass fed beef” refers to correcting calculated grass-fed beef’s operational emissions with statistics of the combined sequestration dataset obtained by merging all three individual datasets. These corrected grass-fed beef emissions can be compared with industrial beef and nonbeef alternatives (red and green) yardsticks, as in Fig. 1*A*. (*B*) Bar 1: Operational emissions not corrected for sequestration. Derived from 10^3^ modeled grass-fed beef operational emissions, considering the full range of plausible geographies, herd types, ration composition, and quality. Bars 2 to 4: Operational emissions (bar 1) minus plausible added rangeland sequestration derived from three recent meta-analyses (60, 74, 75). Filled colored regions and whiskers span percentiles 10 to 90 and 5 to 95, respectively.

Using our herd model, we present grass-fed beef total operational CO_2eq_ emission (the 100-y mean radiative forcing-weighted sum of CO_2_, CH_4_, and N_2_O; see *Methods*) as a function of agricultural intensity, represented by its proxy, the mean ME density (in Mcal (kg dry feed)^−1^) of the consumed rations. Fig. 1*A* reveals that total operational emissions of grass-fed beef are either comparable to or—much more often—larger than those of industrial US beef, and well within the range spanned by global beef operations (73). Fig. 1*B* further resolves this, showing that the added emissions of grass-fed beef over industrial beef hold true for three representative ration densities corresponding to extensive low-quality rations (*Left*), intermediate-quality (*Middle*), and semi-intensive grass-feeding operations. As Fig. 1 and Table 1 (*Methods*) show, emissions of pastured beef from extensive ranches on low productivity rangelands (leftmost bar in 1b, ME = 1.9 Mcal kg^−1^) are 40 to 100% higher than industrial beef (3, 8, 31). This is in general agreement with the range spanned by seven published comparisons of emissions of grazed vs. feedlot beef [Fig. 2*A* and Table 2 of (25)], 20 to 52% higher emissions per unit produced beef by grass-based beef. The same also holds for semi-intensive operations on fine grasslands. Fig. 1*B*, *SI Appendix*, Fig. S4, and Table 1 all show that intermediate-quality rations are carbon optimal. Yet, this least carbon-intensive grass-fed beef is still mostly more intensive than industrial beef.

**Table 1.** Numerical values/ranges used throughout this paper

Variable/parameter	Value or range	Units	Refs.
Emissions of industrial U.S. beef	180 to 220	kg CO_2eq_ (kg protein)^−1^	(3, 8)
Emissions of nonbeef alternatives	10 to 80	kg CO_2eq_ (kg protein)^−1^	This paper’s calculations, green in Figs. 1 *A* and 2 *B*
Raw (sequestration uncorrected) operational emissions of simulated grass fed beef	270 to 410	kg CO_2eq_ (kg protein)^−1^	This paper’s calculations, purple in Figs. 1 *A* and 2 *B*
Sequestration uncorrected operational emissions of pastured beef from extensive ranches on low productivity rangelands	300 to 340	kg CO_2eq_ (kg protein)^−1^	This paper’s calculations, leftmost bar in Fig. 1*B* (ME = 1.9 Mcal kg^−1^)
Sequestration uncorrected operational emissions of pastured beef on intermediate quality rations	200 to 240	kg CO_2eq_ (kg protein)^−1^	This paper’s calculations, middle bar in Fig. 1*B* (ME = 2.2 to 2.3 Mcal kg^−1^)
Sequestration uncorrected operational emissions of pastured beef from semi-intensive operations on fine grasslands	270 to 340	kg CO_2eq_ (kg protein)^−1^	This paper’s calculations, rightmost bar in Fig. 1*B* (ME = 13 Mcal kg^−1^)
Mean (median) of Hao et al.	82 (−36)	kg C (ha y)^−1^	(60)
Mean (median) of Qu et al.	45 (48)	kg C (ha y)^−1^	(74)
Mean (median) of Ren et al.	−167 (0)	kg C (ha y)^−1^	(75)
Combined range of above dataset	−5.1 to 3.4	Mg C (ha y)^−1^	
Characteristic productivity of rangeland based grass fed beef	0.077	ha-year (kg beef protein)^−1^	(73)

**Table 2.** Model parameters and their default values

Parameters		
	Meaning	Default value
*f*_b_	Bulls per cow	0.04
*w*_d_	Cow weight	550 kg
*w*_b_	Bull weight	950 kg
*r_r_*	Replacement rate	0.1 y^−1^
*r_f_*	Finishing rate	0.8 cow^−1^ y^−1^
*h*	Culled healthy rate	0.8

In Fig. 1, all parameter values are the shown defaults. In perturbation Monte Carlo runs, they are perturbed randomly uniformly within ± 10 % of the shown values.

Alternatives (Fig. 1*A*, green rectangle), including pork, poultry, cheese, milk, and plant items, are far less carbon intensive than either grass-fed or industrial beef (1*A*, purple and maroon rectangles, respectively), producing only 5 to 35% of the emissions of the least emissive grass-fed beef herds modeled. While this result has been reported before (76–80), to our knowledge, ours is the first study that explores its dependence on agricultural intensity (through its proxy, ration energy density), thus demonstrating explicitly its validity to rangeland-based grass-fed beef, where beef production does not displace production of alternative food items for direct human consumption (31, 81, 82).

Total grass-fed beef CO_2eq_ emissions shown in Fig. 1*B* exhibit two conflicting dependencies on rations’ ME. As Fig. 1 hints and *SI Appendix*, Fig. S4 confirms, methane emissions (bottom four filled colored bars) decline strongly with rising ME. This dependence stems directly from the decline in feed intake and the fermentable carbohydrate fraction with rising ME and is codified in authoritative equations (42, 43) (*SI Appendix*). At the same time, increasing ME means serving more fossil energy-dependent, CO2-intensive processed roughage (*Methods*). Because most of the drop in methane emissions with rising ME occurs over 1.8≤ME≤2.2 Mcal (dry kg)^−1^, the combination of the two opposing trends yields the rising of total CO_2eq_ emissions Fig. 1 and *SI Appendix*, Fig. S4 display for ME ≳ 2.2. The choice of running a given grass-fed beef operation closer to the extensive (ME → 1.9 Mcal kg^−1^) or semi–intensive (ME ≈ 3 Mcal kg^−1^) operational ends of this continuum is thus also a respective choice between higher overall operational emissions with a higher CH_4_ fraction vs. modestly lower overall operational emissions with a lower CH_4_ fraction (83).

### Effect of Sequestration Enhancement on Total Operational Emission Estimates.

While the results presented in Fig. 1 are consistent with earlier work (49, 84, 85), they disregard sequestration. Can soil carbon sequestration enhancement by grazing cattle reduce or even reverse grass-fed beef’s high emissions? To answer this, we now calculate how estimates of putative sequestration enhancement by grazing cattle (*SI Appendix*, section S3) modify the results of Fig. 1. To do so robustly, we shift the focus from raw operational emissions incurred during production of grass-fed beef to net (sequestration corrected) emissions, defined as these raw operational emissions minus added carbon uptake by the land the cattle occupy that can be credited to the ecological effects of cattle grazing. We also augment the deterministic calculations presented in Fig. 1 and *SI Appendix*, Fig. S4–S5, in which each bar reflects a single ration and the default set of model parameters, with statistical results that consider a wide range of herd types across relevant geographies (*Methods*), summarized in Fig. 2. Because cattle grazing can enhance or undermine sequestration, net emissions can be correspondingly lower or higher than raw operational emissions.

We rely on three 2024 meta-analyzes of carbon uptake on grazed lands (60, 74, 75) to estimate the distribution of sequestration enhancement by cattle grazing. Our most likely estimate of net emissions due to grass feeding is, therefore, calculated by subtracting these estimated empirical distributions, or their unified superdistribution, from the full distribution of simulated operational emissions of grass-fed beef presented in Fig. 1. This range is presented in Fig. 2*A* (*Left* bar), where it can be compared with emissions of industrial US beef and of the combined 5-item nonbeef alternatives. This comparison reveals our key result: Observed emissions per kg protein of industrial beef are lower than about 90% of the simulated grass-feed beef net (sequestration corrected) emissions.

Fig. 2*B* provides further detail. Bar 1 shows the raw (sequestration uncorrected) operational emissions of grass-fed beef (Table 1). Consistent with earlier work (2, 3, 11), these emissions are 20 to 130% higher than emissions of industrial US beef and 3 to 43 times the emissions of the non-beef alternatives described above; see Figs. 1*A* and 2*A* and Table 1.

Bars 2 to 4 of Fig. 2*B* present the operational emissions (Bar 1) after correcting for estimates of sequestration additions by cattle grazing (60, 74, 75) as described in the caption and Methods. The means and medians of the sequestration additions are all small departures from zero relative to the much larger respective ranges (Table 1), and correcting for sequestration makes little qualitative difference: Net emissions of grass-fed beef spanning a wide range of operations, rations, and plausible sequestration all exceed those of industrial beef. After correcting 1,000 simulated herds for observed levels of added sequestration (60, 74, 75), only ≈ 10 to 20% of estimates feature net grass-fed beef emissions lower than those of US industrial beef.

How much added sequestration is required for grass-fed beef to achieve carbon parity with industrial beef? Substituting 10^3^ Monte Carlo emission differences between sequestration uncorrected grass fed beef and industrial beef into Eq. **1** (*Methods*) reveals that carbon parity requires added sequestration of ≈ 430 [240, 740] kg C (ha y)^−1^ (percentiles 50 (5, 86) of the distribution). Can cattle grazing be reasonably expected to elevate sequestration rates by 430 [240, 740] kg C (ha y)^−1^ beyond ungrazed rates on a large scale?

State of the art meta-analyses (29, 60, 74, 75) and an extensive literature review of individual papers (*SI Appendix*, section S8) shows highly variable (87–90) and not infrequently negative (57, 90–92) effects of grazing cattle on soil carbon. The mean and median added carbon sequestration (sequestration under grazing minus sequestration under no grazing) in the 510 matched pairs from widely varied environments reported by the three meta-analyses (60, 74, 75) used in Fig. 2, roughly −170 to 80 kg C (ha y)^−1^, are well below even the lower bound of added sequestration required for carbon parity, 240 kg C (ha y)^−1^. This is further corroborated by another recent meta-analysis (29), as we detail in the SI. Moreover, the pairs compared in Fig. 2 exclude heavy- or over-grazing, known sequestration antagonists, thus likely overestimating sequestration. Even with this stringent exclusion, grazing is often associated with reduced soil carbon stocks. Where grazing enhance carbon sequestration in true rangelands [which do not include the Midwest (31), see, e.g., Fig. 1 of (74)], it rarely does so at sustainable rates exceeding 250 to 300 kg C (ha y)^−1^ (36). Averaging over the large variability, one estimate of achievable global mean grassland sequestration is 100 to 175 kg C (ha y)^−1^ (53), also below the estimated lower bound for carbon parity of 240 kg C (ha y)^−1^.

## Discussion and Conclusions

Our quantification of sequestration-corrected emissions of grass-fed beef shows that, with realistic rangeland sequestration rates, the carbon intensity of most grass fed beef operations is in most cases higher than that of industrial beef, and much higher than that of nonbeef alternatives (Figs. 1 and 2). We also find that among grass-feeding beef operations, the most extensive grazing operations emit the most (Fig. 1 and *SI Appendix*, Fig. S4), for which the modeling framework affords a mechanistic explanation. Grass-fed beef’s methane emissions are high in extensive semiarid, marginal rangeland ranches because such operations are characterized by low energy density rations and decline with rising ration quality, ME density, and agricultural intensity. However, for richer rations with ME≳2.2 Mcal (dry kg)^−1^, the rise of nonmethane emissions (mostly as CO_2_ from fossil energy) outpaces these declines, resulting in rising total CO_2eq_ emissions with rising ME beyond 2.2 Mcal (dry kg)^−1^ (Fig. 1 and *SI Appendix*, Fig. S4).

These results may not apply in low-income nations, where grazing cattle play more existential roles, but this paper focuses on the wealthy nations with high per capita emissions that dominate food and feed production and trade, and thus the global food system. Our results thus have global implications.

These results (Figs. 1 and 2, and *SI Appendix*, Fig. S4) augment earlier findings (22, 29) by showing that, compared with nonbeef alternatives, grass-fed beef yields at most one-tenth of the protein per kg CO_2eq_ emitted regardless of agricultural intensity (and, thus, implicitly, geography). Importantly, the carbon balance perspective is reinforced by the land use one in that the low productivity of grass-fed beef also holds on a per cropland ha basis, as discussed in *SI Appendix*, section S10. Both perspectives synergistically lead to the conclusions (further expanded in *SI Appendix*, section S11) that beef—extensive, intensive, or anything in between—is not a competitive form of resource use because it yields only 1 to 3% of the protein that alternatives do per kg CO_2eq_ emitted or per cropland ha. If food security and ecosystem functioning are the key concerns, rangelands should therefore most sensibly be rewilded, providing important nature-based carbon sequestration and biodiversity benefits, unless they can be rigorously shown to sustainably augment naturally occurring sequestration by at least 240 kg C and realistically by no less than 400 kg C (ha y)^−1^, which very few rangelands do.

The broader corollaries of the findings of this paper can be illustrated by considering the following two hypothetical allocation scenarios for the agricultural resource whose finality imposes the firmest ceiling on food production, arable land.

### Scenario 1.

Addresses extensive beef ranching on semiarid (true) rangelands. There are 8 US states (all western) with state- and annual mean precipitation in the 250 to 500 mm range. Below 250 mm y^−1^, primary productivity is too low for most rainfed ranching, while above 500 mm y^−1^, rainfed production of food for direct human consumption is possible. Jointly, these states comprise ≈ 120 million ha of grassland, pasture, and range land (66). Assuming relatively low productivity befitting of semiarid rangeland of 0.24 to 0.46 ha-y (kg beef protein)^−1^ (73), these lands can supply the US population with 11 to 21 g beef (person day)^−1^ or ≈ 270 to 520 million kg beef protein y^−1^ nationwide, roughly 20 to 40% of recent mean American beef intake. Assuming correspondingly relatively high emissions characteristic of rations dominated by low-quality forage, 345 to 520 kg CO_2eq_ (kg beef protein)^−1^ (73), producing the above amount of beef would require nationwide emissions of ≈ 135 to 270 billion kg CO_2eq_ y^−1^. Now suppose we choose to discontinue this rangeland-based beef production, rewilding 120 million rangeland ha and freeing up the associated cropland also required for this beef production pathway, ≈ 12 million ha, instead. If we choose to use this cropland only to produce as much plant-based protein for human consumption as the forgone beef supplies, 270 to 520 million kg y^−1^, it would allow rewilding of ≈ 120 to 130 million ha (of which ≲ 10% is cropland), along with its associated carbon sequestration and biodiversity benefits, and save annual emissions of 85 to 195 million metric ton CO_2eq_.

### Scenario 2.

Focuses on cropland-based industrial beef production (93–95). US states with statewide annual mean precipitation ≥ 500 mm jointly encompass 128 million ha of grassland and pasture (66). Agriculturally, these croplands can be repurposed from beef grazing to producing most any plant-based food for direct human consumption. Choosing again to use only as much of this cropland as is required for exactly replacing the protein that the forgone beef supplies, in this case, ≈ 82 to 148 g beef (person day)^−1^), about 140 to 260% of current mean intake) or 2 to 3.6 billion kg beef protein y^−1^ nationwide, would save annual emissions of ≈ 260 to 400 million metric ton CO_2eq_ and free 50 to 120 million ha of cropland.

The idea of replacing cropland-based intensive beef grazing with producing nonbeef alternatives may appear to contradict another cropland-focused idea, replacing intensive feed cropping with beef grazing (51, 86, 93, 94, 96, 97). In fact, the ideas are complementary, premised on distinct goals and emphases. Jackson emphasizes (51, 86, 93) croplands’ current degraded state, which is an established consequence of intensive cropping with little attention to nutrient retention or topsoil sparing. As the Introduction describes, our focus, instead, is maximizing croplands’ yield of human-destined food (31, 56, 67) subject to the cattle eat what humans cannot principle that is commonly invoked to support grass feeding.

Returning to the two reallocation scenarios, their comparison makes clear that while both require less land and emissions, replacing cropland-based beef with plant alternatives (Scenario 2) is far more environmentally lucrative (freeing more cropland and emitting less) than forgoing rangeland-based beef (Scenario 1). Modeling productivity and emissions of beef herds using authoritative governing equations thus lends no support to the notion that grass-fed beef is less carbon intensive than industrial beef.

## Methods

### Essential, Widely Used Numerical Ranges.

#### Model description.

Our principal tool, the beef herd model, uses the industry standard US National Academies equations (43) for predicting beef cattle dry matter (DM) feed intake, mean daily weight gain, feed energy requirements for maintenance (DMI, ADG, and NE_mr_, respectively), and CH_4_ emissions of all animals as a function of age, sex, body weight, and ration characteristics. See *SI Appendix*, sections S1 and S2 for further details.

**Fig. 3.**
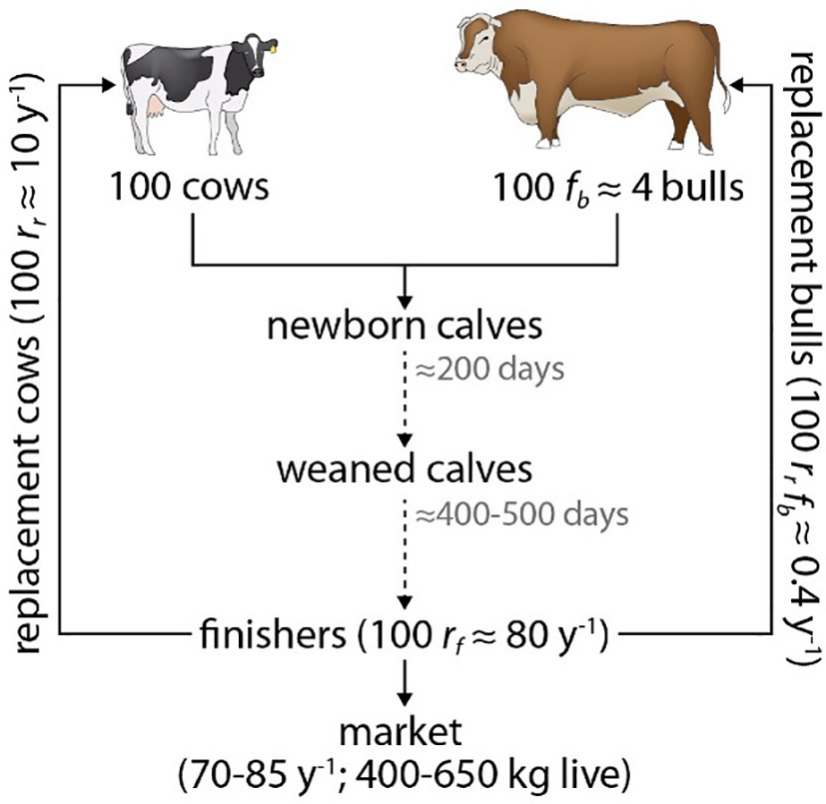
Schematic structure of the modeled herd. See also Tables 1 and 2.

Using this model, we simulate herds of 100 cows and their related reproductive and replacement animals (bulls, replacement heifers, and replacement bulls), as well as unweaned calves and finishing steers (≤2 y old, the herd’s primary beef contributors), diagrammed in Fig. 3. The model parameters and default (unperturbed) values are given in Table 1, and the symbolic beef fluxes in Table 2.

Because feed intake depends on body mass, body mass depends on weight gain, and weight gain depends on feed intake, these three state variables are mutually nonlinearly coupled for growing animals as they evolve in time with a daily time step. Conversely, we assume that the annual mean weights of mature reproductive stock animals are roughly constant, permitting solving their governing equations once for the full year while still observing the nonlinear coupling. *SI Appendix*, section S1 and Supplementary code provide detailed descriptions of the model, the herd it models, and its beef production.

Annual beef yields depend on the fecundity of the cows and the rate at which calves and steers grow when eating the prescribed rations.

#### Representing agricultural intensity using rations’ ME.

We represent the extensive vs. semi-intensive (but still grass dominated) endmembers (e.g., Nevada rangeland vs. Michigan prairie turned heavily managed grassland) using mean ME per kg DM of the modeled animals’ rations, the nutritional energy after accounting for ingested energy loss in liquid, solid, and gaseous excreta in Mcal (kg DM)^−1^. This choice utilizes the observation (98–101) that extensive beef ranches use primarily low productivity true rangelands unfit for other types of agriculture, while semi-intensive grass fed beef operations rely heavily on fine grasslands, with forage quality roughly tracking the rangeland–grassland continuum. For example, while the crude protein dry mass content of native range forage is 8% over the full year, and under 4% in winter (43), for extensively grazed wheat or vegetative fescue, it spans 18 to 22%. Similarly, while the ME content of native range is 2.1 Mcal (dry kg)^−1^ on an annual mean basis, and under 1.8 Mcal (dry kg)^−1^ in winter, it is 2.3 to 2.6 Mcal (dry kg)^−1^ for extensively grazed wheat or vegetative fescue (Table 3).

**Table 3.** Beef fluxes in the model

Subpopulation	Live mass y^1^	Assumed weight
Culled mature animals	100 *r_r_ h* (*w*_d_+*f*_b_*w*_b_)	*w*_d_, *w*_b_
Culled growing animals	100 (1 + *f*_b_) *r_r_* ^2^ *h*/(1−*r_r_*)	2nd y mean live weight
Finishers	100 *r_f_*	min(650 kg, live weight at 2 y)

Based on the above variability in forage energy and protein density, the choice to represent agricultural intensity using ME reflects the dominance of processed forage (hay, silage) of the rations, because such feeds are on average more energy dense than grazed forage (*SI Appendix*, section S1). Complementing Fig. 1 and *SI Appendix*, Figs. S1 and S2 show emissions and feed intake for 15 instead of 3 levels of ME density levels. All rations comprise 90% forage and 10% byproducts by mass (reflecting byproducts’ 10% average contribution to US beef rations (5); sensitivity to those choices is explored later and summarized in Fig. 2). The most meager rations (left bar) represent extensive ranches on arid to semiarid rangelands, while the richest ration (right bar) represents semi-intensive grazing in relatively lush locales.

#### The distinct bars in Fig. 1.

The above details yield the horizontal axis of Fig. 1. The rightmost beef bar in a (ME = 3 Mcal (dry kg)^−1^) corresponds to rich, high-quality rations characteristic of semi-intensive grass-feeding operations that use fine grasslands, while the leftmost bar represents meager rations characteristic of extensive minimum input rangeland-based operations. Because all simulated herds are somewhat extensive and the fraction *f_m_* of total CO_2eq_ emissions due to methane declines with intensity as proportions of highly fermentable carbohydrates in the rations decline from left to right, mean total emissions (total bar heights) are based on—following figure S7i of ref. 73—*f_m_* of (L to R) 0.85, 0.74, and 0.45, with ranges of [0.8,0.9], [0.7,0.8], and [0.4,0.5].

#### A note on CO_2eq_.

We are well aware of the limitations of CO_2eq_ (102), the equivalent CO_2_ mass that would have produced the same century mean radiative forcing as an analyzed mass of non-CO_2_ greenhouse gases. Nonetheless, to facilitate comparisons with earlier studies, we still use CO_2eq_, using the IPCC GWP100 conversion factors (61).

#### Deriving sequestration corrected emissions (Fig. 2’s bars 2 to 4).

Sequestration corrected (net) emissions are given by $E_{net}=E_{operation}-\Delta S\times 0.077\times 44/12$, where $E_{operation}$ denotes total emissions incurred during the production process (Fig. 2*A*, Bar 1), ΔS is added sequestration due to grazing in kg C (ha y)^−1^, 0.077 (Table 1) is median rangeland productivity of grass-fed beef, and 44/12 converts kg C (kg beef protein)^−1^ to kg CO_2eq_ (kg beef protein)^−1^. We estimate added sequestration rates due to grazing using three 2024 relevant meta-analyses (60, 74, 75) of differences in soil organic carbon (SOC) stocks under no vs. light grazing [the grazing pressure least likely to significantly reduce SOC (59), Δ *S* $=\left({\mathrm{SOC}}_{\mathrm{light}\mathrm{grazing}}-{\mathrm{SOC}}_{\mathrm{no}\mathrm{grazing}}\right)/d$ in units of kg C ha^−1^ y^−1^.

While ref. 74 includes information on duration *d*, refs. 60 and 75 do not. For the compared pairs in these datasets, we thus apply d≈10 y. Note that because *d* appears in the denominator of Δ *S*, the larger it is, the smaller Δ *S* becomes. Our 10 y choice—which is half of the mean duration a 2024 US analysis (103) found, and only about 70% of the 14 y mean in (74)—thus generously estimates mean added sequestration rates, quite possibly over emphasizing the environmental benefits of grass fed beef.

#### Monte Carlo populations.

Fig. 2 is based on statistics of *N_mc_* = 10^3^ distinct 100-cow herds subsisting on rations of approximately 70% grazed forage, 20% served processed forage, and 10% byproducts. With these ration defining characteristics (e.g., mean ME=2.1±0.09 Mcal (kg DM)^−1^), this ensemble summarizes 10^3^ slightly distinct implementations (reflecting variability among herds) of a scenario similar to one of bars in the left-middle of *SI Appendix*, Fig. S4), i.e., rangeland-based, rather extensive beef herds, with adequate processed forage additions that account for the months during which grazing is minimal or absent. To further represent seasonal, geographic, and management variability, modeled herds consume rations with random combinations of byproducts, grazed forage, and processed forage, with basic parameters (e.g., replacement rates, weaning rates, bulls per cow) as well as the 70:20:10 feed type proportions all randomly perturbed by ±10%. These simultaneous randomizations ensure that in general, each of the 10^3^ herds is unique, and thus that their statistics are widely representative. Fig. 3 and Tables 1 and 2 describe these parameters and give their default (unperturbed) values. The feed items in the grazed forage, processed roughage, and byproduct portions of the rations are also randomized in individual model runs, thus sampling unique rations that utilize unique combinations of the items available in each category.

#### Estimating the added sequestration needed for grass fed beef carbon parity with industrial beef.

This requisite rate of added sequestration is

[1] $$\Delta S=\left(\frac{12\;\text{kg}\;C}{44\;\text{kg}\;{\text{CO}}_{2}}\right)\frac{\left(\left[270,410\right]-\left[180,220\right]\right)\text{kg}\;{\text{CO}}_{2\text{eq}}{\left(\text{kg}\;\text{protein}\right)}^{-1}}{0.077\left(\text{ha}\times y\right){\left(\text{kg}\;\text{protein}\right)}^{-1}},$$

where the numerator is the emission difference between grass fed beef uncorrected for sequestration and industrial beef, 0.077 ha-year (kg beef protein)^−1^ is the characteristic productivity of rangeland based grass fed beef (73), and ΔS is in kg C (ha y)^−1^. In the Monte Carlo context, the above distribution ranges are replaced by random individual draws from them.

#### Estimating beef non-CH_4_ emissions.

Fig. 1 and 2 address total emissions, the sum of the individual contributions of CH_4_, CO_2_, and N_2_O. Yet, while we calculate beef’s methane emissions explicitly by the beef model, to get the modeled beef’s total emissions requires estimating the combined CO_2_ and N_2_O contribution. We achieve this by amplifying the explicitly calculated grass-fed beef methane emissions of each Monte Carlo realization by dividing it by $f_{{CH}_{4}}$, the methane’s CO_2eq_ fractional contribution (e.g., $f_{{CH}_{4}}=0.6$ if emitted methane mass times methane Global Warming Potential (61, 104) accounts for 60% of beef’s total CO_2eq_ emissions). With $E_{i}^{{CO}_{2eq}}$ and $E_{i}^{{CH}_{4eq}}$ denoting total beef CO_2eq_ emission and methane contribution to it in the *i*th randomized realization, $E_{i}^{{CH}_{4eq}}=f_{{\mathrm{CH}}_{4},i}E_{i}^{{CO}_{2eq}}$, where the left-hand term is calculated by the beef model for each randomized realization. If $f_{{\mathrm{CH}}_{4},i}$ is known, $E_{i}^{{CO}_{2eq}}=E_{i}^{{CH}_{4eq}}/f_{{CH}_{4},i}$ permits estimating total beef emission in the *i*th realization. While the model does not calculate $f_{{\mathrm{CH}}_{4},i}$, we derive a statistically representative population of its values by digitizing (using the online digitizer https://automeris.io/WebPlotDigitizer/) ranges of the fractions of beef’s total emissions accounted for by methane given in *SI Appendix*, Fig. S7 of (73). This digitization reveals that beef’s methane emission account for roughly 40 to 90% of total emissions. We thus let $f_{{CH}_{4}}$ vary linearly uniformly from 40 to 50 at ME = 1.8 to 80 to 90% at ME = 3. Therefore, we set $f_{{\mathrm{CH}}_{4},i}$ to random draws from the uniform distribution between these bounds, with which any realization is randomly assigned a uniformly probable methane fraction approximately between 44 and 92%. Then, the right-hand side of $E_{i}^{{CO}_{2eq}}=E_{i}^{{CH}_{4eq}}/f_{{\mathrm{CH}}_{4},i}$ is fully known, yielding $E_{i}^{{non-CH}_{4eq}}=E_{i}^{{CH}_{4eq}}\left(1-f_{{CH}_{4},i}\right)/f_{{CH}_{4},i}$, where the left hand term denoted the CO_2_ + N_2_O contribution to total CO_2eq_ emissions. For each randomized realization, we thus obtain 5 values, corresponding to the 5 distribution percentiles. Statistics of such distributions are reported in Fig. 1 and by the dark red bars of Fig. 2.

## Supplementary Material

Appendix 01 (PDF)

Dataset S01 (XLSX)

Dataset S02 (CSV)

Code S02 (M)

Code S01 (TXT)

Code S03 (TXT)

## Data Availability

All study data are included in the article and/or supporting information.

## References

[1] M. Clark, D. Tilman, Comparative analysis of environmental impacts of agricultural production systems, agricultural input efficiency, and food choice. Environ. Res. Lett. **12**, 064016 (2017).

[2] D. Tilman, M. Clark, Global diets link environmental sustainability and human health. Nature **515**, 518–522 (2014). 25383533 10.1038/nature13959

[3] G. Eshel, A. Shepon, T. Makov, R. Milo, Land, irrigation water, greenhouse gas and reactive nitrogen burdens of meat, eggs & dairy production in the United States. Proc. Natl. Acad. Sci. U.S.A. **111**, 11996–12001 (2014). 25049416 10.1073/pnas.1402183111 PMC4143028

[4] G. Eshel, A. Shepon, E. Noor, R. Milo, Environmentally optimal, nutritionally aware beef replacement plant-based diets. Environ. Sci. Technol. **50**, 8164–8168 (2016). 27387141 10.1021/acs.est.6b01006

[5] G. Eshel , A model for “sustainable” US beef production. Nat. Ecol. Evol. **2**, 81–85 (2018), 10.1038/s41559-017-0390-5. 29203916

[6] R. T. Pierrehumbert, G. Eshel, Climate impact of beef: An analysis considering multiple time scales and production methods without use of global warming potentials. Environ. Res. Lett. **10**, 085002 (2015).

[7] A. Shepon, G. Eshel, E. Noor, R. Milo, Energy and protein feed-to-food conversion efficiencies in the US and potential food security gains from dietary changes. Environ. Res. Lett. **11**, 1–8 (2016).

[8] G. Eshel, A. Shepon, T. Makov, R. Milo, Partitioning United States’ feed consumption among livestock categories for improved environmental cost assessments. J. Agric. Sci. **153**, 432–445 (2014).

[9] A. C. Castonguay , Navigating sustainability trade-offs in global beef production. Nat. Sustain. **6**, 284–294 (2023).

[10] M. Springmann , Options for keeping the food system within environmental limits. Nature **562**, 519–525 (2018). 30305731 10.1038/s41586-018-0594-0

[11] W. Willett , Food in the Anthropocene: The EAT–Lancet Commission on healthy diets from sustainable food systems. Lancet, **393**, 447–492 (2019), 10.1016/S0140-6736(18)31788-4. 30660336

[12] Z. Chen , Assessment of regional greenhouse gas emission from beef cattle production: A case study of Saskatchewan in Canada. J. Environ. Manage. **264**, 110443 (2020). 32217321 10.1016/j.jenvman.2020.110443

[13] S. Li, Y. Qin, J. Subbiah, B. Dvorak, Life cycle assessment of the U.S. beef processing through integrated hybrid approach. J. Clean. Prod. **265**, 121813 (2020).

[14] H. Harwatt, J. Sabaté, G. Eshel, S. Soret, W. Ripple, Substituting beans for beef as a contribution toward US climate change targets. Clim. Change **143**, 261–270 (2017).

[15] A. Shepon, G. Eshel, E. Noor, R. Milo, The opportunity cost of animal based diets exceeds all food losses. Proc. Natl. Acad. Sci. U.S.A. **115**, 3804–3809 (2018). 29581251 10.1073/pnas.1713820115 PMC5899434

[16] S. Clune, E. Crossin, K. Verghese, Systematic review of greenhouse gas emissions for different fresh food categories. J. Clean. Prod. **140**, 766–783 (2017).

[17] D. L. Katz, K. N. Doughty, K. Geagan, D. A. Jenkins, C. D. Gardner, Perspective: The public health case for modernizing the definition of protein quality. Adv. Nutr. **10**, 755–764 (2019), 10.1093/advances/nmz023. 31066877 PMC6743844

[18] K. T. Sanders, M. E. Webber, A comparative analysis of the greenhouse gas emissions intensity of wheat and beef in the United States. Environ. Res. Lett. **9**, 44011 (2014).

[19] C. A. Rotz, S. Asem-Hiablie, S. Place, G. Thoma, Environmental footprints of beef cattle production in the United States. Agric. Syst. **169**, 1–13 (2019).

[20] B. Putman, C. A. Rotz, G. Thoma, A comprehensive environmental assessment of beef production and consumption in the United States. J. Clean. Prod. **402**, 136766 (2023).

[21] D. O’Brien , LIFE BEEF CARBON: A common framework for quantifying grass and corn based beef farms’ carbon footprints. Animal **14**, 834–845 (2020). 31666147 10.1017/S1751731119002519 PMC7283046

[22] P. L. Stanley, J. E. Rowntree, D. K. Beede, M. S. DeLonge, M. W. Hamm, Impacts of soil carbon sequestration on life cycle greenhouse gas emissions in Midwestern USA beef finishing systems. Agric. Syst. **162**, 249–258 (2018).

[23] S. Roy, S. Bagchi, Large mammalian herbivores and the paradox of soil carbon in grazing ecosystems: role of microbial decomposers and their enzymes. Ecosystems **25**, 976–988 (2022).

[24] Z. Zhang , Regrowth strategies of Leymus chinensis in response to different grazing intensities. Ecol. Appl. **30**, e02113 (2020). 32112460 10.1002/eap.2113

[25] D. F. Cusack , Reducing climate impacts of beef production: A synthesis of life cycle assessments across management systems and global regions. Glob. Chang. Biol. **27**, 1721–1736 (2021). 33657680 10.1111/gcb.15509 PMC8248168

[26] D. D. Briske , Rotational grazing on rangelands: Reconciliation of perception and experimental evidence. Rangel. Ecol. Manage. **61**, 3–17 (2008).

[27] S. Mosier , Adaptive multi-paddock grazing enhances soil carbon and nitrogen stocks and stabilization through mineral association in southeastern U.S. grazing lands. J. Environ. Manage. **288**, 112409 (2021). 33827025 10.1016/j.jenvman.2021.112409

[28] J. E. Rowntree , Ecosystem impacts and productive capacity of a multi-species pastured livestock system. Front. Sustain. Food Syst. **4**, 544984 (2020).

[29] T. Wang, U. Kreuter, C. Davis, S. Cheye, Climate impacts of alternative beef production systems depend on the functional unit used: Weight or monetary value. Proc. Natl. Acad. Sci. U.S.A. **121**, e2321245121 (2024). 39008689 10.1073/pnas.2321245121 PMC11295046

[30] N. E. Tichenor, C. J. Peters, G. A. Norris, G. Thoma, T. S. Griffin, Life cycle environmental consequences of grass-fed and dairy beef production systems in the Northeastern United States. J. Clean. Prod. **142**, 1619–1628 (2017), 10.1016/j.jclepro.2016.11.138.

[31] N. Pelletier, R. Pirog, R. Rasmussen, Comparative life cycle environmental impacts of three beef production strategies in the Upper Midwestern United States. Agric. Syst. **103**, 380–389 (2010).

[32] C. D. Lupo, D. E. Clay, J. L. Benning, J. J. Stone, Life-cycle assessment of the beef cattle production system for the northern great plains USA. J. Environ. Qual. **42**, 1386–1394 (2013). 24216416 10.2134/jeq2013.03.0101

[33] K. R. Heflin, Life–cycle greenhouse–gas emissions of five beef production systems typical of the southern high plains. Agricul. Syst. **176**, 102674 (2015).

[34] J. L. Capper, Is the grass always greener? comparing the environmental impact of conventional, natural and grass-fed beef production systems Animals **2**, 127–143 (2012). 26486913 10.3390/ani2020127 PMC4494320

[35] R. Zhang, J. Wang, S. Niu, Toward a sustainable grazing management based on biodiversity and ecosystem multifunctionality in drylands. Curr. Opin. Environ. Sustain. **48**, 36–43 (2021).

[36] X. Díaz de Otálora , Regenerative rotational grazing management of dairy sheep increases springtime grass production and topsoil carbon storage. Ecol. Indic. **125**, 107484 (2021).

[37] L. Lei , Soil hydrological properties’ response to long-term grazing on a desert steppe in inner Mongolia. Sustainability **15**, 16256 (2023).

[38] B. Wróbel, W. Zielewicz, M. Staniak, Challenges of pasture feeding systems—opportunities and constraints. Agriculture **13**, 974 (2023).

[39] M. N. Hayek , Opportunities for carbon sequestration from removing or intensifying pasture-based beef production. Proc. Natl. Acad. Sci. U.S.A. **121**, e2405758121 (2024). 39495926 10.1073/pnas.2405758121 PMC11572965

[40] K. O. Reinhart, H. S. Sanni Worogo, M. J. Rinella, L. T. Vermeire, Livestock increase soil organic carbon in the Northern great plains. Rangel. Ecol. Manage. **79**, 22–27 (2021).

[41] K. O. Reinhart, M. J. Rinella, R. C. Waterman, H. S. Sanni Worogo, L. T. Vermeire, Carbon sequestration uncertainty: Is grazing-induced soil organic carbon accrual offset by inorganic carbon loss? Rangel. J. **46**, RJ24006 (2024).

[42] P. Escobar-Bahamondes, M. Oba, K. A. Beauchemin, Universally applicable methane prediction equations for beef cattle fed high- or low-forage diets. Can. J. Anim. Sci. **97**, 83–94 (2016). 10.1017/S175173111600121X 27364619

[43] NASEM, National Academies of Sciences, Engineering, and Medicine, Nutrient Requirements of Beef Cattle: Eighth Revised Edition (The National Academies Press, 8th Ed., 2016), 10.17226/19014. 38386771

[44] M. Herrero , Biomass use, production, feed efficiencies, and greenhouse gas emissions from global livestock systems. Proc. Natl. Acad. Sci. U.S.A. **110**, 20888–20893 (2013). 24344273 10.1073/pnas.1308149110 PMC3876224

[45] T. Fetzel, P. Havlik, M. Herrero, K.-H. Erb, Seasonality constraints to livestock grazing intensity. Glob. Chang. Biol. **23**, 1636–1647 (2017). 27976453 10.1111/gcb.13591

[46] K. Wei , Optimizing nitrogen and phosphorus application to improve soil organic carbon and alfalfa hay yield in alfalfa fields. Front. Plant Sci. **14**, 1276580 (2024). 38312359 10.3389/fpls.2023.1276580 PMC10835404

[47] J. L. Capper, The environmental impact of beef production in the United States: 1977 compared with 2007. J. Anim. Sci. **89**, 4249–4261 (2011). 21803973 10.2527/jas.2010-3784

[48] J. F. Soussana , Carbon cycling and sequestration opportunities in temperate grasslands. Soil Use Manage. **20**, 219–230 (2004).

[49] M. N. Hayek, R. D. Garrett, Nationwide shift to grass-fed beef requires larger cattle population. Environ. Res. Lett. **13**, 084005 (2018).

[50] D. O’Brien, M. Markiewicz-Keszycka, J. Herron, Environmental impact of grass-based cattle farms: A life cycle assessment of nature-based diversification scenarios. Resour. Environ. Sustain. **14**, 100126 (2023).

[51] R. D. Jackson, Grazed perennial grasslands can match current beef production while contributing to climate mitigation and adaptation. Agric. Environ. Lett. **7**, e20059 (2022).

[52] R. Lal, Negative emission farming. J. Soil Water Conserv. **76**, 1A–64A (2021).

[53] R. Lal, Digging deeper: A holistic perspective of factors affecting soil organic carbon sequestration in agroecosystems. Glob. Chang. Biol. **24**, 3285–3301 (2018). 29341449 10.1111/gcb.14054

[54] K. Lorenz, R. Lal, “Carbon Sequestration in Grassland Soils BT - Carbon Sequestration” in Agricultural Ecosystems, K. Lorenz, R. Lal, Eds. (Springer International Publishing, 2018), pp. 175–209.

[55] J. F. Soussana , Full accounting of the greenhouse gas (CO2, N2O, CH4) budget of nine European grassland sites. Agric. Ecosyst. Environ. **121**, 121–134 (2007).

[56] C. M. Godde , Soil carbon sequestration in grazing systems: Managing expectations. Clim. Change **161**, 385–391 (2020).

[57] J. Sanderman, T. Hengl, G. J. Fiske, Soil carbon debt of 12,000 years of human land use. Proc. Natl. Acad. Sci. U.S.A. **114**, 9575–9580 (2017). 28827323 10.1073/pnas.1706103114 PMC5594668

[58] Y. Wang , Effect of cultivation history on soil organic carbon status of arable land in northeastern China. Geoderma **342**, 55–64 (2019).

[59] S. Ren , Projected soil carbon loss with warming in constrained Earth system models. Nat. Commun. **15**, 102 (2024). 38167278 10.1038/s41467-023-44433-2 PMC10761705

[60] X. Hao, J. Yang, S. Dong, F. He, Y. Zhang, The influence of grazing intensity on soil organic carbon storage in grassland of China: A meta-analysis. Sci. Total Environ. **924**, 171439 (2024). 38438023 10.1016/j.scitotenv.2024.171439

[61] IPCC, Climate Change 2013 – The Physical Science Basis - Group I Contribution to the Fifth Assessment Report of the Intergovernmental Panel on Climate Change. (2013). 10.1017/CBO9781107415324.

[62] NASS, United States Department of Agriculture, QuickStats (NASS, USDA, 2022).

[63] U. Fao FAOSTAT, Food Agriculture Data (U. Fao FAOSTAT, 2023).

[64] N. R. Haddaway , How does tillage intensity affect soil organic carbon? A systematic review. Environ. Evid. **6**, 30 (2017).

[65] S. Kasa, US trade policy power and sustainable consumption: Beef and cars in North East Asia. J. Consum. Policy **26**, 75–100 (2003).

[66] C. Nickerson, A. Borchers, United States Department of Agriculture, Economic Research Service - How Is Land Used. (Economic Research Service, U.S. Department of Agriculture, 2012).

[67] R. H. Skinner, C. J. Dell, Yield and soil carbon sequestration in grazed pastures sown with two or five forage species. Crop Sci. **56**, 2035–2044 (2016).

[68] C. D. Gardner, J. C. Hartle, R. D. Garrett, L. C. Offringa, A. S. Wasserman, Maximizing the intersection of human health and the health of the environment with regard to the amount and type of protein produced and consumed in the United States. Nutr. Rev. **77**, 197–215 (2019). 30726996 10.1093/nutrit/nuy073 PMC6394758

[69] G. Eshel, P. Stainier, A. Shepon, A. Swaminathan, Environmentally optimal, nutritionally sound, protein and energy conserving plant based alternatives to U.S. Meat. Sci. Rep. **50**, 8164–8168 (2019). 10.1038/s41598-019-46590-1 PMC6687707 31395893

[70] A. A. Musicus , Health and environmental impacts of plant-rich dietary patterns: A US prospective cohort study. Lancet Planet. Heal. **6**, e892–e900 (2022). 10.1016/S2542-5196(22)00243-1 PMC9674022 36370727

[71] J. Derner, G. E. Schuman, Carbon sequestration and rangelands: A synthesis of land management and precipitation effects. J. Soil Water Conserv. **62**, 77–85 (2007).

[72] R. Seager, , Whither the 100th meridian? the once and future physical and human geography of America’s arid–humid divide. Part I: The story so far. Earth Interact. **22**, 1–22 (2018).31097909

[73] J. Poore, T. Nemecek, Reducing food’s environmental impacts through producers and consumers. Science **360**, 987–992 (2018). 29853680 10.1126/science.aaq0216

[74] Q. Qu , Belowground C sequestrations response to grazing exclusion in global grasslands: Dynamics and mechanisms. Agric. Ecosyst. Environ. **360**, 108771 (2024).

[75] S. Ren , Historical impacts of grazing on carbon stocks and climate mitigation opportunities. Nat. Clim. Chang. **14**, 380–386 (2024).

[76] D. Pimentel, Climate changes and food supply. Public Policy Forum Appl. Res. **8**, 54–60 (1993).

[77] D. Pimentel, M. Pimentel, Food, Energy, and Society (University Press of Colorado, 1996).

[78] D. Pimentel, Food, energy, and climate change. Food-Climate Interact. Proc. Work. Berlin **1980**, 303–323 (1981).

[79] D. Pimentel, Energy and agriculture. Food Clim. man. **33**, 199–207 (1979), 10.2480/agrmet.33.199.

[80] G. Eshel, P. A. Martin, Diet, energy, and global warming. Earth Interact. **10**, 1 (2006).

[81] S. I. Apfelbaum , Vegetation, water infiltration, and soil carbon response to Adaptive multi-paddock and conventional grazing in southeastern USA ranches. J. Environ. Manage. **308**, 114576 (2022). 35101805 10.1016/j.jenvman.2022.114576

[82] J. E. Rowntree , Potential mitigation of midwest grass-finished beef production emissions with soil carbon sequestration in the United States of America. Futur. Food J. Food, Agric. Soc. **4**, S31–S38 (2016).

[83] G. Eshel, Planetary Eating (MIT Press, 2025).

[84] M. N. Hayek , A novel correction for biases in forest eddy covariance carbon balance. Agric. For. Meteorol. **250–251**, 90–101 (2018).

[85] M. Hayek, Missing the grassland for the cows: Scaling grass-finished beef production entails tradeoffs—Comment on “Grazed perennial grasslands can match current beef production while contributing to climate mitigation and adaptation”. Agric. Environ. Lett. **7**, e20073 (2022).

[86] R. D. Jackson, Reply to “Missing the grassland for the cows: Scaling grass-finished beef production entails tradeoffs—Comment on ‘Grazed perennial grasslands can match current beef production while contributing to climate mitigation and adaptation’”. Agric. Environ. Lett. **7**, e20082 (2022).

[87] R. T. Conant, C. E. P. Cerri, B. B. Osborne, K. Paustian, Grassland management impacts on soil carbon stocks: A new synthesis. Ecol. Appl. **27**, 662–668 (2017). 27875004 10.1002/eap.1473

[88] L. M. Cardenas , CO2 fluxes from three different temperate grazed pastures using Eddy covariance measurements. Sci. Total Environ. **831**, 154819 (2022). 35346701 10.1016/j.scitotenv.2022.154819

[89] K. Lajtha, L. Silva, Grazing cattle, well-managed or not, is unlikely to increase soil carbon sequestration. Proc. Natl. Acad. Sci. U.S.A. **119**, e2203408119 (2022). 35858462 10.1073/pnas.2203408119 PMC9335331

[90] Y. Bai, M. F. Cotrufo, Grassland soil carbon sequestration: Current understanding, challenges, and solutions. Science **377**, 603–608 (2022). 35926033 10.1126/science.abo2380

[91] L. Deng, Z.-P. Shangguan, G.-L. Wu, X.-F. Chang, Effects of grazing exclusion on carbon sequestration in China’s grassland. Earth-Sci. Rev. **173**, 84–95 (2017).

[92] G. Song, M. Li, P. Fullana-i-Palmer, D. Williamson, Y. Wang, Dietary changes to mitigate climate change and benefit public health in China. Sci. Total Environ. **577**, 289–298 (2017). 27802883 10.1016/j.scitotenv.2016.10.184

[93] R. D. Jackson, America’s dairy grassland – wisconsin milk production that regenerates people and land. Agroecol. Sustain. Food Syst. **48**, 898–915 (2024).

[94] Y. Rui , Persistent soil carbon enhanced in Mollisols by well-managed grasslands but not annual grain or dairy forage cropping systems. Proc. Natl. Acad. Sci. U.S.A. **119**, e2118931119 (2022). 35145033 10.1073/pnas.2118931119 PMC8851490

[95] C. L. Dietz, R. D. Jackson, M. D. Ruark, G. R. Sanford, Soil carbon maintained by perennial grasslands over 30 years but lost in field crop systems in a temperate Mollisol. Commun. Earth Environ. **5**, 360 (2024).

[96] G. Eshel, Small-scale integrated farming systems can abate continental-scale nutrient leakage. PLOS Biol. **19**, e3001264 (2020). 10.1371/journal.pbio.3001264 PMC8174726 34081691

[97] G. Eshel, Small-scale integrated farming systems can abate continental-scale nutrient leakage. PLOS Biol. **19**, e3001264 (2021). 34081691 10.1371/journal.pbio.3001264 PMC8174726

[98] R. L. Naylor, Energy and resource constraints on intensive agricultural production. Annu. Rev. Energy Environ. **21**, 99–123 (1996).

[99] C. Nickerson , Trends in U.S. Farmland Values and Ownership (USDA, 2012).

[100] NASS/USDA, Land Values 2021 Summary (NASS/USDA, 2021).

[101] NASS/USDA, 2018 Agricultural Land, Land Values and Cash Rents (NASS/USDA, 2018).

[102] J. Lynch, M. Cain, R. Pierrehumbert, M. Allen, Demonstrating GWP*: A means of reporting warming-equivalent emissions that captures the contrasting impacts of short- and long-lived climate pollutants. Environ. Res. Lett. **15**, 044023 (2020). 32395177 10.1088/1748-9326/ab6d7e PMC7212016

[103] U. Khatri-Chhetri , Adaptive multi-paddock grazing increases mineral associated soil carbon in Northern grasslands. Agric. Ecosyst. Environ. **369**, 109000 (2024).

[104] B. Z. Masson-Delmotte , “Climate change 2021: The physical science basis” in IPCC, 2021 (2021), 10.1017/9781009157896.

