# Supplementary material for: US grass-fed beef is as carbon intensive as industrial beef and ≈10-fold more intensive than common protein-dense alternatives
Source: Proc Natl Acad Sci U S A. 2025 Mar 17;122(12):e2404329122. doi: 10.1073/pnas.2404329122 (PMC11962457; doi:10.1073/pnas.2404329122)
Supplement: Supplementary file 1 — Appendix 01 (PDF) [file pnas.2404329122.sapp.pdf]

Supporting Information for

U.S. grass fed beef is as carbon intensive as industrial beef, and ≈10-fold more intensive than common protein equivalent alternatives

Gidon Eshel[1], Avi I. Flamholz, Alon Shepon, Ron Milo

February 12, 2025

[1]Corresponding author: Environmental Science, Bard College, Annandale-on-Hudson NY, US; Email: geshel@gmail.com.

List of Figures

S1 Modeled dams' milk yield as a function of days since calving, using Eqs. 2-7 (p. 3381) of (1). On day 47, maximum milk availability mmx ≈ 7 kg d^{-1} is realized. 8

S2 Time series of full body weight (a,c) and cumulative dry feed intake (b,d) of modeled mixed sex finishers (a,b) and replacement heifers (c,d), assuming two ration qualities, both with 10% byproducts by mass. Years are distinguished by alternating gray shading. The more meager ration (with ME $\approx$ 2.1 Mcal (kg DM)$^{-1}$ and 70% and 20% grazed and served forage by mass) is shown by solid curves. The richer ration (with ME $\approx$ 2.4 Mcal (kg DM)$^{-1}$ and 40% and 50% grazed and served forage) are shown by dashed curves. Heifers' first day of pregnancy is highlighted (c,d) with vertical tickmarks. 11

S3 Nutritional differences between rations characterizing extensive and intensive grass fed beef operations. The bars show statistics of intensive minus extensive ration differences of (from left to right) percent neutral detergent fiber NDF (red; a measure of fermentable, least digestible fiber content, mostly from cell walls), metabolizable energy ME (blue), net energy for maintenance NE_m (green), and net energy for growth NE_g (purple) arising in a population of 1000 randomized ration pairs. The intensive rations comprise 10% (by mass) byproducts, 30% grain, 30% processed forage, and 30% native range grazed forage. The extensive rations comprise 10% byproducts and 90% native range grazed forage. Bar colored portions span 10th-90th percentiles, with the whiskers extending to 5th and 95th percentiles. White squares present median differences. Percentages of the 1000 ration pairs for which the intensive ration is richer in the respective nutritional measure than the extensive one (i.e., for which the difference is positive) are given near the upper whisker (denoted p_+). 17

S4 Finer dependence of operational emissions on ration ME, extending Figure 1 of the main text. Right bars correspond to rich, high quality rations typical of semi intensive beef operations. Left bars represent meager rations characteristic of minimum input rangeland based extensive operations. See the caption of Figure 1 of the main text for pictorial details. Bars report CH_4-based CO_{2eq} emissions by, from the bottom up, cows, heifers (first pregnancy females raised to replace culled cows), bulls + their replacement young males, and finisher steers, the herd's main beef source. Percentages of total emissions by steers are reported near the bar tops. Estimated total CO_{2eq} emissions (taking note of CH_4, N_2O, and CO_2) are given above in blue, derived assuming f_m, the fraction of total emission to which methane accounts, ranging from 0.9 on the left to 0.45-0.55 on the right, where the latter choice is guided by Fig. S7 of Poore and Nemecek 2018 (2). In green are emission statistics of the non beef alternative combinations described earlier in this PDF, showing the median (horizontal thick line), the 10th-90th percentile range (shading), and the 5th-95th percentile range (whiskers). Reddish-pink shading shows the range of emissions characterizing U.S. beef (approximately 180-220 kg CO_{2eq} per kg protein, with details in the caption of Figure 1 of the main text). 21

S5 Dependence of feed intake on ration energy density. See the caption of Fig. 2 of the main text for pictorial details. As in earlier figures, right bars correspond to high quality rations characteristic of intensive operations, while left bars represent meager rations characteristic of extensive, minimum input rangeland based operations. Colors correspond to (from the bottom up) cows, heifers, bulls + their replacement young males, and finisher steers. Percentages of total feed intake for which steers account are reported near bar tops. 22

S6 Distributions of soil organic carbon stock difference ΔSOC between lightly grazed plots and paired comparable ungrazed ones. These distributions are based on recent meta-analyses (3–5) which jointly comprise 510 pairs in widely variable environments with under 850 mm y^{-1} climatological precipitation. While the meta-analyses include lightly-, moderately-, heavily-, and over-grazed sites, we only consider lightly grazed members (in (3, 5)) or lightly or moderately grazed ones (in (4)). Because heavy or over grazing are most likely to undermine soil organic carbon, this choice means that the presented net (sequestration corrected) grass fed beef emissions are conservatively biased toward high sequestration and thus low net emissions. 24

S7 Repeating bars 2 and 4 of Fig. 2 of the main text for the refs. (3, 5) data while assuming the default $d = 10$ y (middle of each 3-bar group, the first and the second of which presenting the ref. (3) and ref. (5) data respectively) or $d = 8$ or 14 y (left and right of those central bars respectively). For comparison with industrial beef, bar 5 of Fig. 2 of the main text, showing the emission range characterizing industrial U.S. beef, is reproduced here in the same color as in Fig. 2 of the main text, with the mean shown by a horizontal line. 25

S8 Dependence of various full herd production and emission attributes on ration density ME. 29

Sections of supporting text:

Contents

S1 The numerical model **7**

S1.1 Governing equations 7

S1.1.1 Growing animals 7

S1.1.2 Dams . . . 10
S1.1.3 Bulls . . . 12
S1.2 Herd structure: Live animal fluxes and standing stocks . . . 12
S1.3 Beef production, edible and protein fractions . . . 14

S2 Model validation 15

S2.1 Against the existing UN-FAO model GLEAM . . . 15
S2.2 Against grass fed beef life cycle assessments . . . 16

S3 The choice of metabolizable energy (ME) to represent the intensive-to-extensive agricultural continuum 16

S4 The mixed item protein conserving alternatives to beef used in Figs. 1 and 2 of the main text 18

S5 Land use information and its contributions to Fig. 2 of the main text 19

S6 Expanding on Figure 1 of the main text 20

S7 Feed mass intake statistics of the simulated herds 20

S8 Review of published estimates of added carbon sequestration attributable to cattle grazing 20

S8.1 The need for sustainable, enduring estimates . . . 23

S8.2 Published per hectare carbon stock and sequestration estimates 23

S8.2.1 The sequestration rates underlying the results Figure 2 23

S8.2.2 Comparison to independent estimates 26

S9 Consistency between our Fig. 2 results and those of Wang et al. 2024 28

S10 Dependence of beef production and CH_4 emissions on ME 29

S11 Land productivity of Elko County, Nevada 29

S12 The consistency and mutual reinforcement of the emission and land use dimensions of the efficiency comparison of grass fed beef and non beef alternatives 30

Accompanying software

The following Matlab (Version: 9.14.0.2306882, R2023a, Update 4) code files accompany this paper:

1. The code that generates Figure 1 of the main text and S10 and S5 of this document is given in `SoftwareS1.m`.
2. The code that generates Figure 2 of the main text is given in `SoftwareS2.m`.
3. The basic beef herd numerical model called by both `SoftwareS1.m` and `SoftwareS2.m` is given in `SoftwareS3.m`.

All code contains copious comments, informal yet unambiguous references, and further explanation, all prefaced (i.e., preceded on the left)—as dictated by the matlab parser and convention—by percent signs.

S1 The numerical model

Below, key terms, defined quantities, and notations are boxed pink.

S1.1 Governing equations

The governing equations predict dry matter intake, mean daily weight gain, and feed energy requirements for maintenance (widely known in animal science as DMI, ADG, and NEm respectively). They are based on the 2016 8th Edition of the National Academies' *Nutrient Requirements of Beef Cattle*, hereafter NASEM16 (6).

Following NASEM16 (6) and traditional animal science notation, we use the following notation, all applied to a particular animal with known characteristics

DMI is Dry Matter Intake in kg dry feed animal^{-1} d^{-1},

FBW is Full Body Weight in kg,

$\mathrm{SBWfac} = 0.960$ is the conversion factor from full to shrunk body weight (6),

$\mathrm{EBWfac} = 0.891$ is the conversion factor from shrunk to empty body weight (6),

$\mathrm{SBW} = \mathrm{SBWfac} \cdot \mathrm{FBW}$ is Shrunk Body Weight in kg (6),

$\mathrm{MW} = \mathrm{SBW}^{0.75}$ is Metabolic Weight in kg$^{0.75}$ (6, eq. 19-1),

$\mathrm{EBW} = \mathrm{SBWfac} \cdot \mathrm{EBWfac} \cdot \mathrm{FBW}$ is Empty Body Weight in kg (6),

$\mathrm{a1} = 0.077$ is base metabolic rate or fasting heat production, Mcal kg$^{-0.75}$ (6).

We assume no environmental stress (which means assuming temperatures in the 15°–25°C range and no hastening of winter heat loss by mud coating).

S1.1.1 Growing animals

We assume $\text{calf birth weight} = 36\ \text{kg}$ (characteristic of Hereford newborns), and consider growth of $\leq$4 y. This upper bound is rarely enacted, because most specified rations permit finishers to

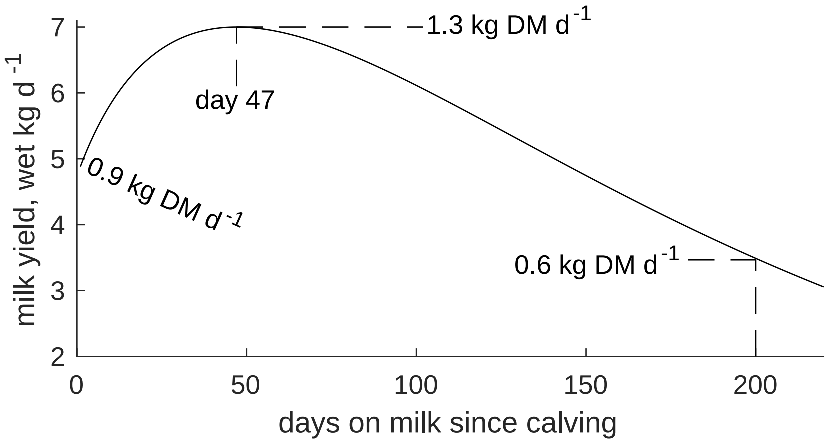

Figure S1: Modeled dams' milk yield as a function of days since calving, using Eqs. 2-7 (p. 3381) of (1). On day 47, maximum milk availability `mmx` $\approx$ 7 kg d^{-1} is realized.

attain market weight, 650 kg, much younger.

Growth of nursing calves (ages 0-220 days, the latter being d_{wean}, weaning day) follow (1, 7), assuming milk dry matter content = 18% (1), and milk energy content of `Em` ≈ 0.7 Mcal (kg milk)$^{-1}$ (which follows from assuming 4% fat and 8% non-fat solids (6, eq. 19-24)).

We model dam milk yield `MYt` using eq. 2-7 (p. 3381) of (1). It is a recasting of the Wood Model, which was found (7) to perform slightly better than NASEM16. Fig. S1 presents the resultant lactation curve.

With milk availability and energy content thus modeled (so that calves' available milk energy is `MYt`·`Em`), we begin the birth-to-weaning time stepping scheme, lines 92-113 in `SoftwareS3.m`, in which `NEma` and `NEga` are the specified ration's density of net energy for maintenance and growth in Mcal (kg DM)$^{-1}$, `NEmr` is the animal's required maintenance net energy in Mcal d^{-1}, `fat` is its % fat content, and the methane calculation (line 112) follows (8).

The logical-numerical sequence of this growth is as follows.

```
daily time-stepping loop for day<=d_wean = [2,220]
   - calculate shrunk, empty and metabolic body weights
   - use body weight and maximum milk availability to calculate
     digestible energy intake and feed intake DMI
```

```
    - use metabolic weight and milk energy availability to calculate
      retained energy
    - use retained energy and empty body weight to calculate daily
      empty and live body weight gain
    - use previous day live weight and daily live weight gain to update
      full body live weight
    - use body weight and feed intake to calculate methane emissions
end daily time-stepping loop
```

Beyond weaning (i.e., for $d > d_{\mathrm{wean}}$), we distinguish the sexes and treat them individually by setting (6) `SEX = 1.15` and 1 for replacement bulls and heifers respectively. Until this split, and for finishers thereafter, we assume an even 50:50 male:female split, thus setting `SEX = 1.075`. We assume no anabolic implants, thus setting `ADTV = 0.94` (6, table 19-6).

Days $d > d_{\mathrm{wean}}$ are treated in lines 121-322 in `SoftwareS3.m`, using the following logical flow.

```
- assign SEX assuming equal mean number of males and females
- assign the appropriate div factor (Eq. 19-90 of NASEM2016)
  used to map metabolic weight onto feed intake DMI
- calculate the metabolic weight to feed intake DMI map for the
  remainder of year 1
- calculate the metabolic weight to feed intake DMI map for age > 1 y
daily time-stepping loop for day > weaning day
    - if 221 < day < 366
          use 0.1128 in NASEM2016 eq 19-88
    - otherwise (if day > 365)
          use 0.0869 in NASEM2016 eq 19-88
    - endif
    - calculate shrunk and metabolic body weights
    - calculate daily feed intake
    - refine daily feed intake based on how close the animal weight is
      to its desired final weight at the expected final fat content
    - calculate the metabolic energy the animal retains
    - use retained energy and equivalent empty body weight to calculate
      daily empty body weight gain
    - use daily empty body weight gain to calculate daily live weight gain
    - use previous day live weight and daily live weight gain to update
      full body live weight
    - use body weight and feed intake to calculate methane emissions
```

`end daily time-stepping loop`

Following (6), we calculate young (pre-conception) heifer's feed intake using shrunk body weight (not its 0.75 power), and forgo the `eqSBW` calculation, as above. This means using `DMI(i) = SBW*DMIfac*ADTV` (6, Eq. 19-92) in which `DMIfac = 0.012425 + .019218*NEma - 0.007259*NEma`^{2} (6, eq. 19-91,92,93) to obtain feed intake, followed by calculating retained energy `RE` (6, eq 19-48). The heifer calculations continue from the day of first conception, the day $d =$ `pregd0` their weight first exceeds $\approx$ 310 kg, which—because it is a strong function of ration quality—is in general unique to each specified ration composition (each bar in Fig. 1 of the main text or in Fig. S10 and S5 of this document).

These calculations for growing animals (heifers, finisher steers, replacement bulls) continue until certain criteria are met. For heifers, the female-specific growth continues over a full term pregnancy, days `pregd0` + [0,280]. Finishers are grown until they first attain market weight, 650 kg. Finally, for replacement bulls, growth continues until their weight approaches 90% of mature bull weight. We present a few example growth curves in Fig. S2.

S1.1.2 Dams

The annual feed intake of a dam combines basic maintenance needs with those of pregnancy and lactation, all functions of shrunk body weight to the 0.75 power (6, Eq. 19-1). We assume dam weight—550 kg—is uniform over the year. Of course this is a simplification—these weights vary over their lactation depending on milk production, pregnancy phase, among other factors—but we assume these weight changes vanish when averaged over a suitably long period.

We distinguish several pregnancy and lactation periods following delivery day. On days 1-85, dams are lactating, but not pregnant. On days 86-178, they are lactating while in their 1st trimester of pregnancy. During days 179-220, pregnancy advances but lactation gradually declines until drying on day 220. Finally, days 221-365 account for the rest of the pregnancy, until next calving on day

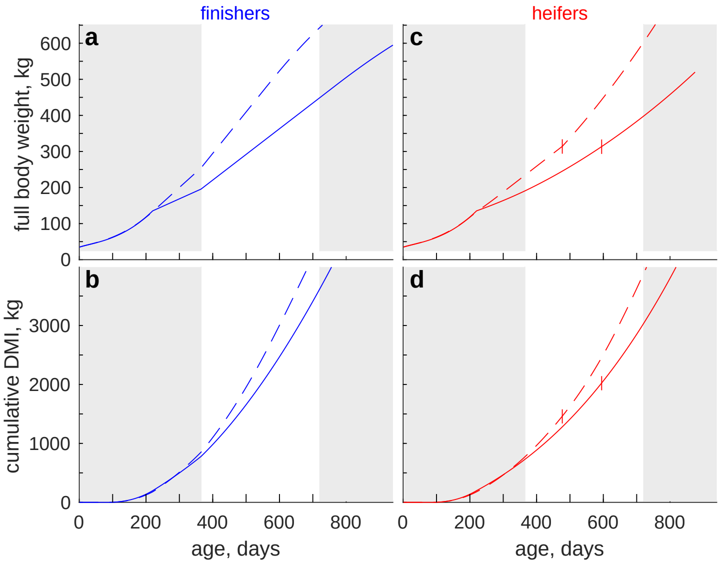

Figure S2: Time series of full body weight (a,c) and cumulative dry feed intake (b,d) of modeled mixed sex finishers (a,b) and replacement heifers (c,d), assuming two ration qualities, both with 10% byproducts by mass. Years are distinguished by alternating gray shading. The more meager ration (with ME $\approx$ 2.1 Mcal (kg DM)$^{-1}$ and 70% and 20% grazed and served forage by mass) is shown by solid curves. The richer ration (with ME $\approx$ 2.4 Mcal (kg DM)$^{-1}$ and 40% and 50% grazed and served forage) are shown by dashed curves. Heifers' first day of pregnancy is highlighted (c,d) with vertical tickmarks.

365. The code segment that addresses this partitioning—lines 378-390 of `SoftwareS3.m`—is based on (6, Eqs. 19-94,95,96), with the final line summing all daily feed intakes during the above phases of the lactation into a scalar holding dams' annual emissions.

S1.1.3 Bulls

Because we again assume time-invariant bull weight, the calculation for bulls—lines 392-397 of `SoftwareS3.m`—is straightforward. Feed intake is

```
DMI = 365*SBW^0.75*a1;
```

in Mcal y^{-1} (6), where $SBW^{0.75}$ (metabolic weight) is multiplied by the basic feed intake `a1 = 0.077` Mcal $kg^{-0.75}$ (6). Annual methane emissions are

```
CH4 = 365*(71.5 + 0.12*FBW + DMI^3 *fat3 );
```

as before.

S1.2 Herd structure: Live animal fluxes and standing stocks

We envision a herd in annual mean steady state, maintaining its annual mean size, with $N_c = 100$ cows and $N_b = N_c f_b$ bulls (see same table), which defaults to 4 bulls (9). We assume replacement/failure rates of $r_r = 0.1$ (table 1 of the main text) or 10% per year. The herd structure is shown schematically in Fig. 3 of the main text. The numbers of growing young animals—fattened finishers, and replacement heifers and bulls—depend on the quality of the offered rations.

Because we assume a default dam culling rate of $N_c r_r$ cows y^{-1}, which in the absence of a perturbation defaults to $100 \cdot 0.1$, steady state requires ≈ 10 new heifers to enter the herd annually.

To maintain this flux, N_0 female calves enter the heifer pool, undergo the usual attrition, eventually reaching their final number N_e. Given replacement rate r_r y^{-1} (which we assume mirrors failure rate), the initial heifer number becomes $N_0(1-r_r)$ after one year, and $N_0(1-r_r)^\tau$ for any arbitrary maturation period of τ years (where τ need not be a natural number). Because growth is slower on meager rations, τ is much longer on those rations, e.g., 3.4, 2.2, and 2.1 years for ME of 1.9, 2.3, and 3 Mcal (dry kg)$^{-1}$. These rather disparate τs amount to a key difference among the various bars in Figs. 1,2, S5 and S10. For each specific ration (either deterministically prescribed as in Figs. S5 and S10 or in Fig. 1 of the main text, or randomized, as in the calculations summarized in Fig. 2 of the main text) heifer maturation period is $\tau =$`length(FBW.hfr)/365`, where `FBW.hfr` is the array holding the heifers' weight evolution, whose length (number of elements) is the number of days it takes heifers to reach first conception weight (which defaults to $\approx$ 310 kg), plus 280 day pregnancy. With τ thus obtained and attrition rate r_r, the final heifer number after τ years is $N_e = N_0(1-r_r)^\tau$, as given above. For calculating feed intake and methane emissions, we use the mean number of heifers in the herd,

$$N_m := \frac{1}{2}\left(N_0 + N_e\right) = \frac{N_0}{2}\left[1 + (1-r_r)^\tau\right]. \tag{S1}$$

For herd continuity, the final heifer number, at age τ years, must equal the required number of new heifers annually, i.e.,

$$N_e = N_c r_r \quad\rightarrow\quad N_0(1-r_r)^\tau = N_c r_r \quad\rightarrow\quad N_0 = \frac{N_c r_r}{(1-r_r)^\tau}, \tag{S2}$$

which yields roughly 12, 13, and 14 required starting heifers per 100 dams for $\tau = 2$, 2.6, and 3.2 years. With these N_0, the mean heifer number is

$$N_m = \frac{N_c r_r\left[1 + (1-r_r)^\tau\right]}{2(1-r_r)^\tau}, \tag{S3}$$

112, 116, and 120 heifers for a 1000 dam herd for the same τ values.

Note that a very similar range is obtained if we instead approach attrition continuously. Then,

$dN/dt = -r_r N$, whose solution is trivially $N_\tau = N_0 e^{-r_r \tau}$. Equating this to the required annual flux of entering heifers yields $N_0 e^{-r_r \tau} = N_c r_r$ or $N_0 = N_c r_r e^{r_r \tau}$, with which the mean heifer standing stock is

$$N_m = \frac{1}{2}\left(N_0 + N_e\right) = \frac{N_c r_r}{2}\left(e^{r_r \tau} + 1\right), \tag{S4}$$

111, 115, and 119 heifers for a 1000 dam herd for the same τ values.

We apply similar logic and corresponding notation to replacement bulls, from which we derive the number and flux of finishers,

$$N_{\text{finishers}} = N_c(1 - r_r) - N_{\text{heifers}} - N_{\text{replacement bulls}}, \tag{S5}$$

where the first right hand term is (a generous estimate of) the approximate annual calf recruitment rate, 90 newborns per year per 100 dams. For the variable rations considered in Figs. S5 and S10, finisher numbers are approximately 77-78 annually.

S1.3 Beef production, edible and protein fractions

By far the dominant beef source—accounting for 85-90% of total beef output—arises from finisher steers. The remainder 10-15% of total beef output arises from animal culling, assumed (as described earlier) 10% per year of each demographic group, of which we assume 80% are healthy enough to yield marketable beef. The most important flux of this type arises from culled cows, 100 dams $\times$ 550 kg of live weight dam^{-1} $\times$ 0.1 y^{-1} culling rate $\times$ 0.8 healthy fraction = 4400 kg live weight beef per year.

For the variable rations considered in Figs. S5 and S10, the total live weight beef output of the 100 dam herd is roughly 35-55 metric tons annually

To transform live weight into edible beef, we assume 56% dressing rate and 75% deboned portion, yielding overall live-to-edible fraction of 42% (10, 11). To then convert edible beef mass to beef

protein, we assume 20% protein content of edible beef, i.e.,

$$\text{annual beef protein output} := \text{annual live weight beef output} \times 0.56 \times 0.75 \times 0.2, \tag{S6}$$

i.e., yielded beef protein mass is 8.4% of the yielded live weight beef mass.

S2 Model validation

S2.1 Against the existing UN-FAO model GLEAM

GLEAM is one of several models—such as Carbon Audit, CAP'2ER and Bovid-CO2—that quantify spatially explicit cradle to farm gate carbon footprint of various livestock on regional, national, or global scales (as discussed by, e.g., (12)). All use agreed upon animal science equations such as the NASEM ones we use (or their CSIRO or other counterparts outside of the U.S.) to evaluate performance, needs, and emissions of individual herd members are the same ones we use.

One source of GLEAM based results is a 2013 FAO report (13), available online at `https://www.fao.org/4/i3461e/i3461e.pdf`. In their Fig. 8, they report beef emissions of 28-29 and 40-45 kg CO_{2eq} (kg carcass)$^{-1}$ for temperate and arid locales respectively (an important distinction, especially in key North American grazing grounds (14)). While the U.S. is mostly temperate, parts of the west and southwest qualify as arid. Assuming that this represents 20-25% of U.S. beef and that carcass weight $\approx$ 55-65% live weight, and applying the above factor of 8.4% protein in live weight, the most probable weighted average range of their GLEAM based emission intensities that's appropriate for U.S. beef is 200-250 kg CO_{2eq} (kg beef protein)$^{-1}$. This partly overlaps with our results (Fig. 1), expansively 220-330 kg CO_{2eq} (kg beef protein)$^{-1}$. The 25-30% discrepancy both makes sense and is expected given that we model pure grass based ranches while the GLEAM calculations merely envision $>$ 10% of the dry matter fed to animals is farm produced, which can and often does also include processed roughage (hay, silage) with far higher average nutritional profile than rangeland grass.

S2.2 Against grass fed beef life cycle assessments

Tichenor et al. 2017 (15) applied life cycle assessment to northeastern U.S. dairy and beef farms, and found mean emissions of 33.7 kg CO_{2eq} (kg hot carcass weight)$^{-1}$. With their reported 54% dressing rate, and assuming 75% bone free fraction in the dressed beef and 20% protein by mass in the deboned fraction, this amounts to about 410-420 kg CO_{2eq} (kg beef protein)$^{-1}$, in good agreement with Figs. 1 and 2 of the current paper's main text.

Analyzing data from a pasture based farm in Devon, England, McAuliffe et al. 2018 (16) reported (their Fig. 4) a 12-31 kg range of CO_{2eq} (kg live beef)$^{-1}$, or—using the same factors as above—150-375 kg CO_{2eq} (kg beef protein)$^{-1}$, expanding somewhat but otherwise in good agreement with the range spanned by Fig. 1 and the leftmost bar of Fig. 2 of the main text.

S3 The choice of metabolizable energy (ME) to represent the intensive-to-extensive agricultural continuum

As discussed in the main text, we use metabolizable energy (ME, the nutritional energy after accounting for losses of ingested energy via gaseous, liquid and solid excreta, in Mcal per kg dry matter feed, Mcal (kg DM)$^{-1}$) as a proxy for agro-practical intensity. "Agro-practical intensity" locates a given ranch along a continuum between two endmembers. One addresses extensive ranches that use rangelands and favor minimizing inputs and labor, thus attaining low productivity. The other is the opposite, addressing intensive, high productivity ranches that use generously inputs and labor on a mixture of cropland and fine grassland. The choice of the ME proxy is based on the general relationship between operation intensity and the richness of cattle rations in those operations (measured by. e.g., g protein or kcal per kg feed), with the two rising in concert; the higher the intensity, the richer the rations. To be sure, the intensive–extensive dichotomy is not perfectly mirrored by such nutritional attributes as ME. For example, at over 2.5 Mcal (kg DM)$^{-1}$, spring mixed native rangeland forage is in fact richer than a typical high quality

served forage, alfalfa hay (2.2 Mcal (kg DM)$^{-1}$), and just as rich as another, corn silage (6). This calls into question using ME for representing the operational intensity differences between the above two endmembers. Despite these exceptions, we nonetheless use ME to represent these differences because considering rations as a whole, mean differences between the intensive and extensive rations are large and consistent enough to render ME a robust proxy for location along the intensive–extensive continuum, justifying the choice.

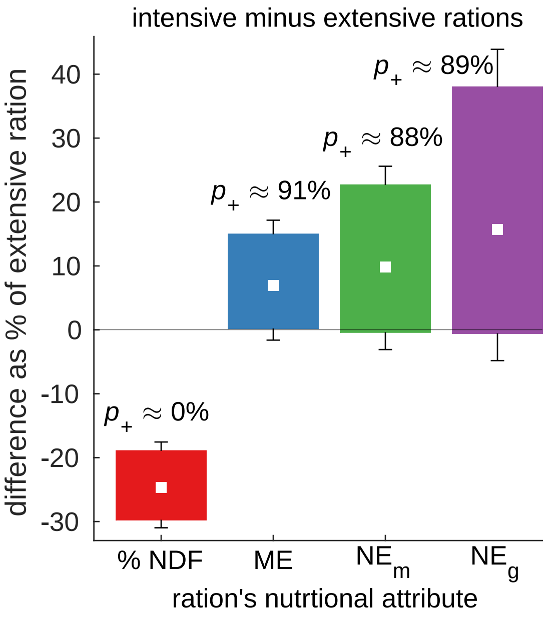

Figure S3: Nutritional differences between rations characterizing extensive and intensive grass fed beef operations. The bars show statistics of intensive minus extensive ration differences of (from left to right) percent neutral detergent fiber NDF (red; a measure of fermentable, least digestible fiber content, mostly from cell walls), metabolizable energy ME (blue), net energy for maintenance $\mathrm{NE_m}$ (green), and net energy for growth $\mathrm{NE_g}$ (purple) arising in a population of 1000 randomized ration pairs. The intensive rations comprise 10% (by mass) byproducts, 30% grain, 30% processed forage, and 30% native range grazed forage. The extensive rations comprise 10% byproducts and 90% native range grazed forage. Bar colored portions span 10th-90th percentiles, with the whiskers extending to 5th and 95th percentiles. White squares present median differences. Percentages of the 1000 ration pairs for which the intensive ration is richer in the respective nutritional measure than the extensive one (i.e., for which the difference is positive) are given near the upper whisker (denoted p_+).

Demonstrating this, Figure S3 shows that when considering statistics of numerous realistic rations of the two agro-practical endmembers, all nutritional measures considered are heavily lopsided. All extensive rations' % NDF (a measure of the least digestible, highly fermentable components in

forage) exceed those of their intensive counterparts. Consistently, the energy density of $\approx 90\%$ of the intensive rations exceeds those of the extensive ones, with median differences of 7, 10 and 16% for the three energy measures shown. The figure thus shows that in extensive operations, animals must consume (if the bulk of the ingested fresh feed permits) more feed per unit available energy, on account of rations richer in such relatively poorly digestible cell wall structural carbohydrates as lignin or cellulose. This is hardly surprising, because forages are well-known to be rich in these fermentable complex carbohydrates, but Figure S3 shows that—as expected—the digestive job of the animals in extensive operations is harder than in the intensive ones.

These chemical differences are further amplified by differences in required grazing efforts. For a 550 kg animal, e.g., grazing is estimated to require about 2.1-2.6 Mcal d^{-1} of energy beyond its non-grazing maintenance energy requirements (17, 18). While this $\approx 20\%$ range is not inconsequential, it is not unexpected (19, 20) for such highly variable phenomena that strongly depend on widely variable weather and topographical conditions, and on forage utilization that can reasonably vary over 25-35% (21–23). Because the base maintenance energy requirements of such an animal are about 8-9 Mcal d^{-1}, the above estimates of 2.1-2.6 Mcal d^{-1} of additional requirements due to grazing amount to a quarter to a third higher needs of grazing animals.

Because extensive operations' rations differ substantially from those of intensive operations in energy attributes that greatly impact productivity, and because these differences are coherent and robust, ME serves as a useful practical proxy for agro-practical intensity, the proxy we use for modeling various beef operations ranging in intensity.

S4 The mixed item protein conserving alternatives to beef used in Figs. 1 and 2 of the main text

In addition to presenting beef resource needs, Figs. 1 and 2 of the main text also show (in green) resource use by non-beef, protein conserving mixed alternative "diets". We calculate $N_{mc} = 10^3$

such Monte Carlo realizations, typically indexed in following equations by i. Each comprises 5 non-beef alternative items which may include animal items—pork, poultry, eggs, cheese, milk—as well as such plant items as rice, wheat, oats, legumes, nuts and peanuts, tubers, among others. Because all beef results are reported per kg protein, protein conservation simply means that the alternative items also jointly deliver 1 kg protein. For simplicity, and with some loss of generality, we assume that the 5 included items contribute equal protein masses, 200 g item^{-1}.

The pth emission percentile ($p = [5, 10, 50, 90, 95]$) of the ith alternative "diet" is $E_i^p = \frac{1}{5}\sum_{j=1}^{5} e_{j(i)}^p$, where $e_{j(i)}^p$ is the data based emissions (2) of the jth randomly chosen alternative item in combination i (i.e., j is a distinct set for each i combination). Using E_i^p and e_j^p in kg $CO_{2\text{eq}}$ (kg protein)$^{-1}$ ensures protein conservation, with replaced beef and its mixed item alternatives both delivering 1 kg protein. The green elements in Figs. 2 and 3a,b of the main text show statistics of E_i^p.

S5 Land use information and its contributions to Fig. 2 of the main text

The calculations Fig. 2 summarizes require total land use and the rangeland and cropland portions thereof. While total use data are available from (2), we are unaware of any comprehensive data on the rangeland:cropland partitioning. We thus estimate this partitioning by assuming the 1:9 cropland:rangeland areal split we found to characterize U.S. beef (24, 25). This assumption is reasonable—we are primarily focused on developed nations—but non-universal, thus limiting the generality of this paper. We apply the 1:9 split to beef's total land use data, whose distribution (percentiles 5, 10, 50, 90, and 95) are available from (2). We denote the pth distribution percentile of total land use by the jth of 5 food items randomly selected in the ith "diet" $L_{j(i)}^p$ ha-y (kg protein)$^{-1}$, which reduces to L_b^p for the 1-item beef "diet". We use these distribution percentiles (2) in Fig. 2 of the main text in two ways, as follows.

The beef bars of Fig. 2 show statistics of $E_b^p = e_{\mathrm{b},i}/(0.9L_b^{pn})$, beef emissions per unit rangeland used, where—consistent with earlier notation—$e^p_{b,i\in[1,N_{mc}]}$ denotes the pth distribution percentile of total operational emissions of the ith realization of modeled grass fed beef in kg $CO_{2\mathrm{eq}}$ (kg beef protein)$^{-1}$. Note that by explicitly using all distribution percentiles in the denominator above regardless of where along the spectrum of possible values an individual beef emission realization (the numerator) falls, we expansively sample the combinatorial space emissions and land use jointly form. This yields 5 E_b^p kg $CO_{2\mathrm{eq}}$ (rangeland ha y)$^{-1}$ values for each Monte Carlo beef realization (or $5N_{mc}$ values overall), whose distribution is shown by the red bars in Fig. 2.

In a corresponding non beef manner, the green bar in Fig. 2 shows emission statistics of the alternative "diets", i.e., of the N_{mc} values of E_i^p introduced above.

S6 Expanding on Figure 1 of the main text

In the main text, Fig. 1 presents only 3 ration qualities. Yet our calculations are much more finely resolved in ME density. Fig. S10 presents these more resolved results.

S7 Feed mass intake statistics of the simulated herds

Because environmental burdens of beef depend on feed intake, Fig. S5 presents feed intake statistics for the simulated herds.

S8 Review of published estimates of added carbon sequestration attributable to cattle grazing

Here we address the most ubiquitously invoked counterargument to beef's outsized environmental liabilities, the possibility that grazing enhances carbon sequestration by grasslands (26–28) enough

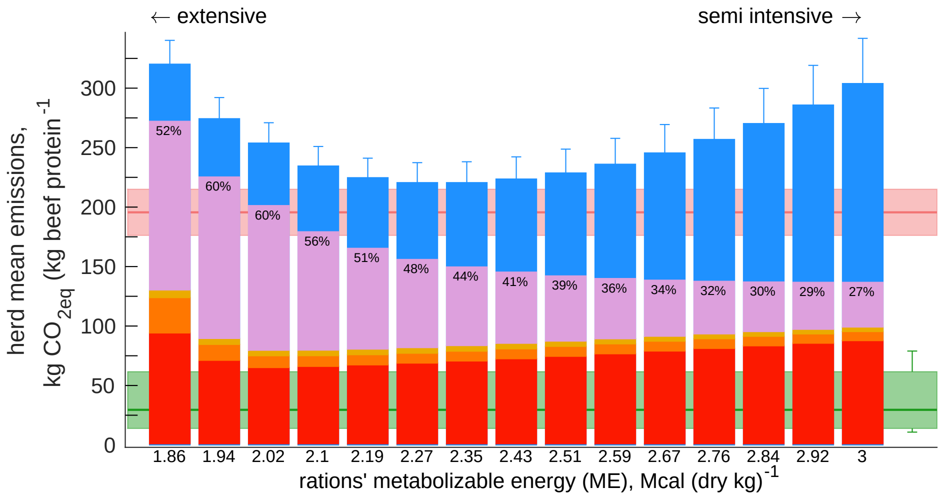

Figure S4: Finer dependence of operational emissions on ration ME, extending Figure 1 of the main text. Right bars correspond to rich, high quality rations typical of semi intensive beef operations. Left bars represent meager rations characteristic of minimum input rangeland based extensive operations. See the caption of Figure 1 of the main text for pictorial details. Bars report CH_4-based CO_{2eq} emissions by, from the bottom up, cows, heifers (first pregnancy females raised to replace culled cows), bulls + their replacement young males, and finisher steers, the herd's main beef source. Percentages of total emissions by steers are reported near the bar tops. Estimated total CO_{2eq} emissions (taking note of CH_4, N_2O, and CO_2) are given above in blue, derived assuming f_m, the fraction of total emission to which methane accounts, ranging from 0.9 on the left to 0.45-0.55 on the right, where the latter choice is guided by Fig. S7 of Poore and Nemecek 2018 (2). In green are emission statistics of the non beef alternative combinations described earlier in this PDF, showing the median (horizontal thick line), the 10th-90th percentile range (shading), and the 5th-95th percentile range (whiskers). Reddish-pink shading shows the range of emissions characterizing U.S. beef (approximately 180-220 kg CO_{2eq} per kg protein, with details in the caption of Figure 1 of the main text).

to meaningfully reduce the high emissions of beef production. Of course that photosynthesis takes up radiatively active CO_2 gas and transforms it into belowground radiatively inert carbon forms is not in question. But that this uptake is enhanced by grazing (29, 30) is more conjectural and less certain. In part, this is because such putative enhancements likely depend on geography, climate, and weather, and thus vary widely in time (e.g., following weather variability on 2-10 day timescales, the seasonal cycle, and climate variability on interannual, decadal or longer timescales) and space (e.g., to differ widely among arid, semi arid, or temperate locales).

The argument is promotes in several ways. Some emphasize specific grassland management models, notably "rapid rotational" or "adaptive multi-paddock" grazing (31, 32), as particularly effective at

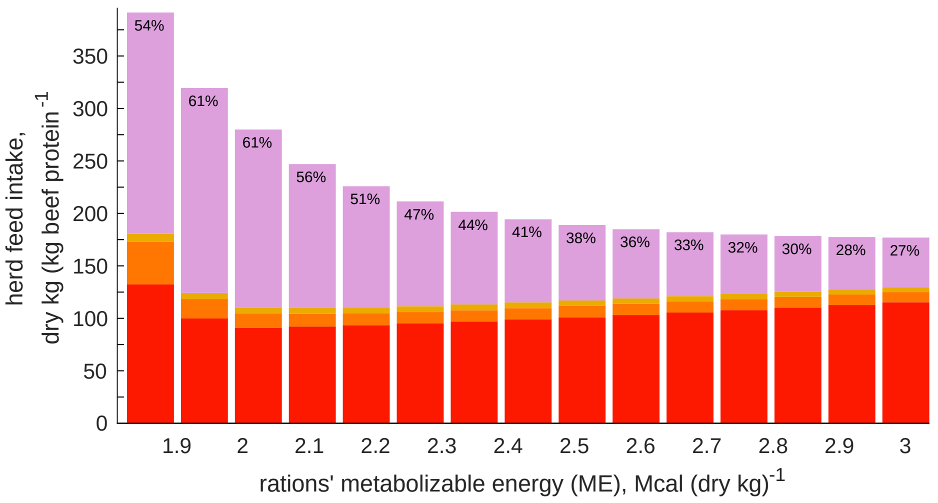

Figure S5: Dependence of feed intake on ration energy density. See the caption of Fig. 2 of the main text for pictorial details. As in earlier figures, right bars correspond to high quality rations characteristic of intensive operations, while left bars represent meager rations characteristic of extensive, minimum input rangeland based operations. Colors correspond to (from the bottom up) cows, heifers, bulls + their replacement young males, and finisher steers. Percentages of total feed intake for which steers account are reported near bar tops.

enhancing grassland carbon sequestration. Others focus on a specific phase of beef production, finishing, in which steers gain 50-65% of their market weight during a brief sprint (33–35). This emphasis recognizes that while conventional finishing is based on concentrated feeds and fine hay and silage, which use valuable high quality cropland and fossil fuel based agrochemicals (synthetic fertilizer, herbicides), it can become grass based instead. But rapid weight gain is essential for profitability, and most genuine (semi arid, marginal) rangelands are too meager for such gains (34, 36). Consequently grass finishing (like conventional finishing) still uses croplands that can produce most alternative food items, but for direct grazing rather than for mechanized production of grains, hay, or silage.

This idea has some observational support. Particularly notable is the particularly careful and compelling modeling study of Midwestern U.S. beef production (36). It shows that whereas feedlot and grass finished beef require emitting about 15 and 19 kg CO_{2eq} per live beef kg produced, assuming substantial but realistic carbon sequestration by the used grazed cropland reduced these values to 13 and 11 kg. Carbon sequestration can thus turn grass finished beef from being 30%

more to 15% *less* carbon intensive than conventional finishing. Yet at ≈140 kg CO_{2eq} (kg beef protein)$^{-1}$, carbon intensity of the produced beef is still 4-5 times higher than that of the non beef alternatives Figs. 1 and 2 of the main text report.

S8.1 The need for sustainable, enduring estimates

Soil carbon tends to saturate with time. This raises the need for sustained, long term observations, because while sequestration may be fast immediately following management improvements, especially in lush temperate grasslands (37–40), after 2-4 decades (41, Figs. 2b and 3) it tends to slow markedly and even vanish (42, Fig. 3), (43). Brief analyses of overused rangelands—whose native perennial vegetation is mostly replaced by invasive annuals (44), whose soils are compacted and organic matter depleted (45), with correspondingly reduced water holding capacity (46) that reduces water availability and promotes soil erosion (47, 48)—are thus not generalizable enough (49–51). Evaluating the potential role of sequestration in mitigating anthropogenic emissions based on brief observations—which is quite common (52, Table 1)—is thus grossly misleading, because these rates are bound to decline (53–56).

S8.2 Published per hectare carbon stock and sequestration estimates

S8.2.1 The sequestration rates underlying the results Figure 2

The observational data on the impact of grazing on soil carbon stocks and uptake rates on which Fig. 2 of the main text is based are three recent meta analyses (3–5). Of those, only one (4), includes data on the duration d of the grazing exclosure experiment. For each of these 124 compared pairs, the added sequestration rate due to light grazing is

$$\Delta S = \frac{\Delta \mathrm{SOC}}{d} \equiv \frac{\mathrm{SOC}_{\text{light grazing}} - \mathrm{SOC}_{\text{no grazing}}}{d} \left[\frac{\text{kg C}}{\text{ha y}}\right], \tag{S7}$$

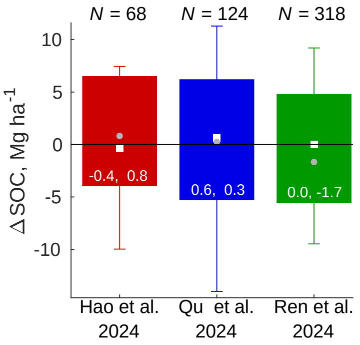

Figure S6: Distributions of soil organic carbon stock difference ΔSOC between lightly grazed plots and paired comparable ungrazed ones. These distributions are based on recent meta-analyses (3–5) which jointly comprise 510 pairs in widely variable environments with under 850 mm y^{-1} climatological precipitation. While the meta-analyses include lightly-, moderately-, heavily-, and over-grazed sites, we only consider lightly grazed members (in (3, 5)) or lightly or moderately grazed ones (in (4)). Because heavy or over grazing are most likely to undermine soil organic carbon, this choice means that the presented net (sequestration corrected) grass fed beef emissions are conservatively biased toward high sequestration and thus low net emissions.

where ΔSOC is the carbon stock difference due to light grazing in kg C ha^{-1}. The three data sets' ΔSOC distributions are given in Fig. S6.

But Fig. 2 of the main text presents operational emissions minus $\Delta S = \Delta \mathrm{SOC}/d$, yet d, the required duration of the exclosure experiments, is only available for the ref. (4) data.

For the pair comparisons in refs. (3, 5), which lack d data, we assume that the duration is the minimum of the mean and median of the ref. (4) data, i.e., set $d = \min\{\mathrm{mean}_{\mathrm{Qu}}, \mathrm{median}_{\mathrm{Qu}}\} = \min\{14, 10\} = 10$ y throughout. While clearly not as robust as relying on observed durations, as in (4), $d = 10$ y is conservative, because only 45% of the (4) durations are under 10 y, and the correction due to sequestration is proportional to $1/d$, so that $10 < 14$ yields a larger sequestration correction and thus lower net emissions. Most importantly, this choice impacts the results quantitatively but not qualitatively, with all 6 median and mean net emissions exceeding even industrial beef's 220 kg CO_{2eq} (kg protein)$^{-1}$ upper bound, as Fig. S7 shows.

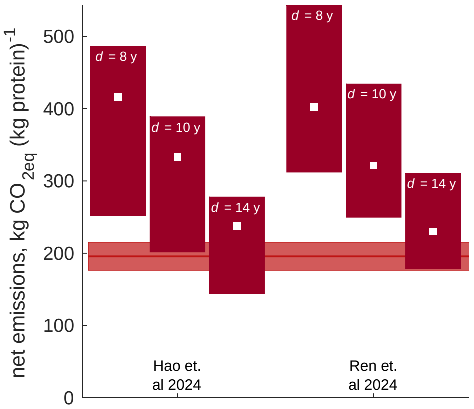

Figure S7: Repeating bars 2 and 4 of Fig. 2 of the main text for the refs. (3, 5) data while assuming the default $d = 10$ y (middle of each 3-bar group, the first and the second of which presenting the ref. (3) and ref. (5) data respectively) or $d = 8$ or 14 y (left and right of those central bars respectively). For comparison with industrial beef, bar 5 of Fig. 2 of the main text, showing the emission range characterizing industrial U.S. beef, is reproduced here in the same color as in Fig. 2 of the main text, with the mean shown by a horizontal line.

S8.2.2 Comparison to independent estimates

A recent analysis (57) of four published comparisons of topsoil organic carbon estimates in native (unaltered) grassland and similar unmanaged plots used for cattle grazing (57, Figure S2 and Table S2) reveals that grazed grasslands have 30-40% lower sequestered soil carbon than native grasslands. Twenty other savanna pairs showed 16-18% lower soil carbon in grazed than in native savannas. These observations thus show that grazing *removes* soil organic carbon, not enhance it.

Similar results also characterize 353 grassland pair comparisons (58), in which the ungrazed/undisturbed sites had about 45% more soil organic carbon than their grazed/degraded counterparts (58, Figure 4b). (But some of these differences are likely due to overgrazing, whose negative impact on SOC is well appreciated.) A similar analysis compared soil organic carbon content of grazed/ungrazed pairs in six depth ranges collectively spanning the top 1.2 m of soil in Kenya (59). In all 6 depths, ungrazed plots had the most organic carbon content, continuously grazed plots had the least, with rotationally grazed plots in between.

One unusually long term (3-4 decade) comparison (60) addressed four sites in a montane, dry

Wyoming sagebrush steppe with exclosures (small fenced plots within the larger site from which grazing is excluded). Only one of the four sites had significant grazed–ungrazed ΔSOC. A 16 year comparison (61) of unmanaged grazed and ungrazed sites in another high elevation, arid rangeland revealed about double the soil organic matter in the ungrazed plots. A 74-y exclosure study in a slightly less arid Colorado shortgrass steppe found that moderately grazed or ungrazed plots had $\Delta\mathrm{SOC} \approx 0$, with no SOC enhancement by grazing. No soil organic carbon response to grazing or its absence was also obtained for a 15-y exclosure–grazed plot comparison in a humid subtropical Florida grassland with $\approx$ 1400 mm y^{-1} (62). A consistent and more extreme result characterized the analysis (63) of 15 grazing exclosures in temperate Inner Mongolia grasslands. There were no soil carbon storage differences in 10-30 cm, with a modest 10% increased carbon storage in the soil top 10 cm, where rapid turnover greatly limits the climate benefits of the added belowground carbon storage.

Consistent results also characterize a 20-y comparison of carbon and nitrogen inputs, outputs and content of various ecosystem components in semi-arid African savannas grazed by various combinations of wild herbivores and cattle (64). Importantly, the studied cattle featured extremely low densities, 5-10 hectare per individual, well below common stocking rates, all but precluding the possibility of overgrazing. Even under these forgiving conditions, cattle removed soil carbon and nitrogen at unsustainable rates, while elephants and other megaherbivores increased these pools and, when cohabiting with cattle the same plots, even reversed the negative effects of cattle. This corroborates a 29-y followup of plant diversity—not a direct measure but a useful yet imperfect proxy of SOC (65)—in a relatively lush U.S. grassland grazed by native bison or cattle (66), which showed plant diversity increasing with time in bison but not cattle grazed sites.

Analysis of 15 grazed–ungrazed pairs in the South American grasslands (67) focused on the more durable, slower to decompose mineral associated soil organic matter. It revealed that while this prized organic carbon form increased in upland (well drained, topographically higher) grazing exclusion sites, the reverse happened in shallow and lowland sites (67, Figure 4a). Similarly, the (56) analysis revealed conflicting impacts of grazing on soil organic carbon, with positive *and*

negative results of widely varying magnitudes apparent from the surface to a depth of 60 cm (56, Figure 4). While they estimated a mean sequestration rate increase due to improved grazing of 280 kg C (ha y)$^{-1}$, this mostly reflects (56, Figure 2) just two comparisons showing large added sequestration under grazing improvements, with the rest showing little soil carbon with or without the improvements, with a vanishing mean difference. Qualitatively similarly mixed results also characterized the analysis (68) of a mixed grass prairie and a (higher and drier) sagebrush steppe in Wyoming, neither used for cattle grazing. While the 5-year mean carbon uptake in the drier site was 300 kg C (ha y)$^{-1}$, very close to the above 280 kg, the less arid, a bit warmer site took up no carbon. This highlights a key explanation for the widely divergent, sometimes contradictory results summarized above: with or without any grassland modification by grazing, grasslands can differ greatly in all attributes, including carbon uptake rates.

Comparisons of grazed and ungrazed neighboring sites thus show that herbivore free grasslands can sustainably thrive, and store as much carbon as most grazed rangelands do. To the extent our limited knowledge permits general conclusions, the response of grassland soil carbon stocks to grazing appears highly variable and sign inconsistent. Grazing may reduce greenhouse gas emissions in locations with good soils and ample precipitation, but most such landscapes can also produce far more food of most any kind (Figs. 1 and 2 of the main text). Using true rangelands for cattle grazing is by no means a reproducible, robust atmospheric carbon sink. It can be, but rangelands' responses to being grazed vary by climate, soil, topography, vegetation characteristics, fire permission or suppression, and resident vs. migratory dominant grazers, among other determinants (69–72). True rangelands can thus realistically sequester—sustainably and on large spatial scales—at most 100-200 kg C (ha y)$^{-1}$, comparable to the sequestration corrections used in Fig. 2 of the main text.

S9 Consistency between our Fig. 2 results and those of Wang et al. 2024

In the Results section, we note that the three added sequestration meta analyses used in generating Figure 2 span $\approx [-170, 80]$ kg C (ha y)$^{-1}$, well below even the lower bound of added sequestration required for carbon parity, 240 kg C (ha y)$^{-1}$. This is further corroborated by another recent meta analysis (73) that is not used in Figure 2 because it reports reduction in carbon intensity due to added sequestration, not these additions themselves. It reports that accounting for C sequestration reduces net emissions by 8.5 kg CO_{2eq} (kg carcass weight)$^{-1}$ or about 17 kg C (kg protein)$^{-1}$. When divided again by rangeland mean productivity (0.077 ha-year (kg beef protein)$^{-1}$, Table 1) this emission reduction yields the underlying added sequestration, 220-225 kg C (ha y)$^{-1}$, also below the lower bound of added sequestration required for carbon parity, 240 kg C (ha y)$^{-1}$.

S10 Dependence of beef production and CH_4 emissions on ME

The purpose of this section is present the plot on which the discussion of the two regimes Figures 1 of the main text and here explained in the *Discussion and Conclusions* section of the main text.

S11 Land productivity of Elko County, Nevada

In the main Discussion and Conclusions, we note that compared with non beef alternatives, grass fed beef yields at most one tenth of the protein per kg CO_{2eq} emitted regardless of agricultural intensity (and, thus, implicitly, geography), and that this also holds true on a per ha of cropland or rangeland basis. Here, we demonstrate the latter point by analyzing USDA data for Elko County, Nevada, a focal center of rangeland based cow–calf operations.

Using USDA data (74), we note that the county has 1.9 million acres or 781 thousand ha of

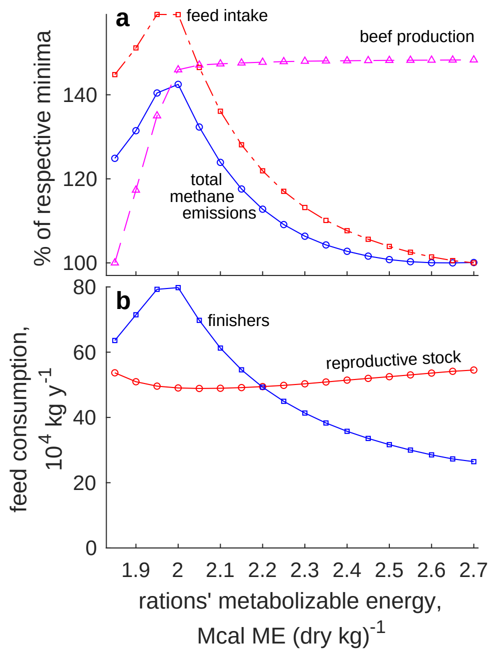

Figure S8: Dependence of various full herd production and emission attributes on ration density ME.

pastureland, and that it sells a total of 79 thousand heads of cattle a year, of which calves and ≥500 lb steers comprise 38% and 62%. Assuming the characteristic sale weight of these two cattle categories is 70 and 260 kg respectively, we get a weighted average sale weight of 190 kg. Multiplying this by the above 79 thousand heads of cattle a year sold yields annual shipments of about 15 million live kg of cattle.

Dividing 15 million live kg of produced cattle by 781 thousand ha of pastureland yields 19.2 kg live weight beef per ha per year. Assuming—as throughout this paper, see earlier and the main text—84 g yielded beef protein per yielded kg live weight, we obtain the final result, 1.6 kg beef protein per rangeland ha.

Elko county, Navada thus yields under 2 kg beef protein (rangeland ha year)$^{-1}$. By comparison, land productivity of the non beef food categories in (74) considered here is $\approx 270 \pm 260$ kg protein (ha y)$^{-1}$. This comparison—2 to 7 vs. 270 ± 260 kg protein (ha y)$^{-1}$ for beef and its alternatives, respectively—shows why the emissions and land use standpoints are mutually consistent and thus amplifying: beef—extensive, intensive, or anything in between—is not a competitive land use

because it yields only 1-3% of the protein alternatives do.

S12 The consistency and mutual reinforcement of the emission and land use dimensions of the efficiency comparison of grass fed beef and non beef alternatives

To quantify this most stringently, let's consider the optimal grass fed beef scenario (Figure 1a, middle bar). In it, producing one kg of beef protein requires 220-240 kg dry feed, of which 18-22% is processed forage, i.e., no less than 40-50 dry kg processed forage. Given U.S. wide rainfed hay yields, 2450 ± 1200 dry kg ha^{-1} y^{-1} (74), producing 40-50 dry kg hay (and thus a kg of grass fed beef protein) requires 0.01-0.03 ha-y of cropland. If reallocated from beef to production of alternatives (that include, recall, dairy, cheese, poultry, pork, eggs, and plant staples, using (2) data), these 0.01-0.03 ha-y of cropland can yield 4.2 [2,10] kg protein while emitting 80-90 kg CO_{2eq} compared to beef's 1 kg protein and 175-200 kg CO_{2eq}. Combining the above differences yields beef emissions of 175-200 kg CO_{2eq} (kg protein)$^{-1}$ compared with the considered alternatives' 20 [8,45].

Correcting these operational emissions for possible offsetting by sequestration enhancement has very limited qualitative impact. Employing the rough 1:9 cropland:rangeland partitioning of land used in beef production (24, 25), 9 times beef's cropland needs yields a rough estimate of 0.1-0.3 ha-y of rangeland needed per kg beef protein (a global estimate (2) is about 0.15). Assuming 200-350 kg C (ha y)$^{-1}$ of added sequestration due to cattle grazing on this needed rangeland yields a possible sequestration offset of 30-140 kg CO_{2eq} (kg beef protein)$^{-1}$. This turns current U.S. beef's operational emissions, 180-220 kg $_{2eq}$ (kg protein)$^{-1}$ (24, 25), into sequestration corrected net emissions of 35-170 kg CO_{2eq} (kg protein)$^{-1}$, still mostly above the alternatives'.

References

[1] Tedeschi, L. O & Fox, D. G. (2009) *Journal of Animal Science* **87**, 3380–3391.

[2] Poore, J & Nemecek, T. (2018) *Science* **360**, 987–992.

[3] Hao, X, Yang, J, Dong, S, He, F, & Zhang, Y. (2024) *Science of The Total Environment* **924**.

[4] Qu, Q, Deng, L, Shangguan, Z, Sun, J, He, J, Wang, K, Zhou, Z, Li, J, & Penuelas, J. (2024) *Agriculture, Ecosystems & Environment* **360**.

[5] Ren, S, Terrer, C, Li, J, Cao, Y, Yang, S, & Liu, D. (2024) *Nature Climate Change* **14**, 380–386.

[6] NASEM. (2016) *Nutrient Requirements of Beef Cattle: Eighth Revised Edition.* (National Academies of Sciences, Engineering, and Medicine (NASEM), The National Academies Press, Washington, DC), 8 edition, p. 2065.

[7] Lancaster, P. A, Tedeschi, L. O, Buessing, Z, & Davis, M. E. (2021) *Journal of Animal Science* **99**, 1–19.

[8] Escobar-Bahamondes, P, Oba, M, & Beauchemin, K. A. (2016) *Canadian Journal of Animal Science* **97**, 83–94.

[9] Thundathil, J. C, Dance, A. L, & Kastelic, J. P. (2016) *Theriogenology* **86**, 397 – 405. Proceedings of the 18th ICAR.

[10] Service, U. E. R. (2021) Food availability (per capita) data system (`https://www.ers.usda.gov/data-products/food-availability-per-capita-data-system/`, note = Accessed January 8 2021).

[11] Coyne, J. M, Evans, R. D, & Berry, D. P. (2019) *Journal of Animal Science* **97**, 1501–1512.

[12] O'Brien, D, Herron, J, Andurand, J, Caré, S, Martinez, P, Migliorati, L, Moro, M, Pirlo, G, & Dollé, J.-B. (2020) *Animal* **14**, 834–845.

[13] Opio, C, Gerber, P, Mottet, A, Falcucci, A, Tempio, G, MacLeod, M, Vellinga, T, Henderson, B, & Steinfeld, H. (2013) Greenhouse gas emissions from ruminant supply chains – a global

life cycle assessment, (Food and Agriculture Organization of the United Nations (FAO), Rome), Technical report. Online, accessed June 21 2024.

[14] Khatri-Chhetri, U, Thompson, K. A, Quideau, S. A, Boyce, M. S, Chang, S. X, Bork, E. W, & Carlyle, C. N. (2024) *Agriculture, Ecosystems & Environment* **369**.

[15] Tichenor, N. E, Peters, C. J, Norris, G. A, Thoma, G, & Griffin, T. S. (2017) *Journal of Cleaner Production* **142**, 1619 – 1628.

[16] McAuliffe, G, Takahashi, T, Orr, R, Harris, P, & Lee, M. (2018) *Journal of Cleaner Production* **171**, 1672–1680.

[17] Brosh, A, Henkin, Z, Ungar, E. D, Dolev, A, Shabtay, A, Orlov, A, Yehuda, Y, & Aharoni, Y. (2010) *Journal of animal science* **88**, 315–323.

[18] Osuji, P. O. (1974) *Journal of Range Management* **27**, 437–443.

[19] Aharoni, Y, Henkin, Z, Ezra, A, Dolev, A, Shabtay, A, Orlov, A, Yehuda, Y, & Brosh, A. (2009) *Journal of animal science* **87**, 2719–2731.

[20] Marco, O. N. D & Aello, M. S. (1998) *Journal of Range Management* **51**, 9–13.

[21] Hervey, J. A. Q & Donald, F. (1970) *Joirnal of Range management* **23**, 50–55.

[22] Galt, D, Molinar, F, Navarro, J, Joseph, J, & Holechek, J. (2000) *Rangelands* **22**, 7–11.

[23] Smart, A. J, Derner, J. D, Hendrickson, J. R, Gillen, R. L, Dunn, B. H, Mousel, E. M, Johnson, P. S, Gates, R. N, Sedivec, K. K, Harmoney, K. R, Volesky, J. D, & Olson, K. C. (2010) *Rangeland Ecology & Management* **63**, 397–406.

[24] Eshel, G, Shepon, A, Makov, T, & Milo, R. (2014) *Proceedings of the National Academy of Sciences* **111**, 11996–12001.

[25] Eshel, G, Shepon, A, Makov, T, & Milo, R. (2014) *The Journal of Agricultural Science* **153**, 432–445.

[26] Rowntree, J. E, Ryals, R, DeLonge, M, Teague, W. R, Chiavegato, M. B, Byck, P, Waang, T, & Xu, S. (2016) *Future of Food: Journal on Food, Agriculture and Society* **4**, S31–38.

[27] Lal, R. (2021) *Journal of Soil and Water Conservation* **76**, 61A–64A.

[28] Cusack, D. F, Kazanski, C. E, Hedgpeth, A, Chow, K, Cordeiro, A. L, Karpman, J, & Ryals, R. (2021) *Global Change Biology* **27**, 1721–1736.

[29] Roy, S & Bagchi, S. (2022) *Ecosystems* **25**, 976–988.

[30] Zhang, Z, Gong, J, Wang, B, Li, X, Ding, Y, Yang, B, Zhu, C, Liu, M, & Zhang, W. (2020) *Ecological Applications* **30**.

[31] Teague, R & Kreuter, U. (2020) *Frontiers in Sustainable Food Systems* **29**.

[32] Mosier, S, Apfelbaum, S, Byck, P, Calderon, F, Teague, R, Thompson, R, & Cotrufo, M. F. (2021) *Journal of Environmental Management* **288**, 112409.

[33] Harmoney, K. R & Jaeger, J. R. (2018) *Kansas Agricultural Experiment Station Research Reports* **4**.

[34] Stanley, P. L, Rowntree, J. E, Beede, D. K, DeLonge, M. S, & Hamm, M. W. (2018) *Agricultural Systems* **162**, 249–258.

[35] McGee, M, Lenehan, C, Crosson, P, O'Riordan, E, Kelly, A, Moran, L, & Moloney, A. (2022) *Agricultural Systems* **198**.

[36] Pelletier, N, Pirog, R, & Rasmussen, R. (2010) *Agricultural Systems* **103**, 380–389.

[37] Soussana, J, Allard, V, Pilegaard, K, Ambus, P, Amman, C, Campbell, C, Ceschia, E, Clifton-Brown, J, Czobel, S, Domingues, R, Flechard, C, Fuhrer, J, Hensen, A, Horvath, L, Jones, M, Kasper, G, Martin, C, Nagy, Z, Neftel, A, Raschi, A, Baronti, S, Rees, R, Skiba, U, Stefani, P, Manca, G, Sutton, M, Tuba, Z, & Valentini, R. (2007) *Agriculture, Ecosystems & Environment* **121**, 121–134.

[38] Godde, C. M, de Boer, I. J. M, zu Ermgassen, E, Herrero, M, van Middelaar, C. E, Muller, A, Roos, E, Schader, C, Smith, P, van Zanten, H. H. E, & Garnett, T. (2020) *Climatic Change* **161**, 385–391.

[39] Wilkinson, J. M, Chamberlain, A. T, & Rivero, M. J. (2021) *Agronomy* **11**.

[40] Hammar, T, Hansson, P.-A, & Roos, E. (2022) *Journal of Cleaner Production* **331**.

[41] West, T. O & Six, J. (2007) *Climatic Change* **80**, 25–41.

[42] Zomer, R. J, Bossio, D. A, Sommer, R, & Verchot, L. V. (2017) *Scientific Reports* **7**.

[43] Tyson, K, Roberts, D, Clement, C, & Garwood, E. (1990) *The Journal of Agricultural Science* **115**, 29–40.

[44] Davies, K. W, Leger, E. A, Boyd, C. S, & Hallett, L. M. (2021) *Journal of Environmental Management* **288**.

[45] Manzano, M. G & Navar, J. (2000) *Journal of Arid Environments* **44**, 1–17.

[46] Basant, S, Wilcox, B. P, Leite, P. M, & Morgan, C. L. (2020) *Environmental Research Letters* **15**.

[47] Kairis, O, Karavitis, C, Salvati, L, Kounalaki, A, & Kosmas, K. (2015) *Arid Land Research and Management* **29**, 360–374.

[48] Teague, W. R, Apfelbaum, S, Lal, R, Kreuter, U. P, Rowntree, J, Davies, C. A, Conser, R, Rasmussen, M, Hatfield, J, Wang, T, Wang, F, & Byck, P. (2016) *Journal of Soil and Water Conservation* **71**, 156–164.

[49] Conant, R. T & Paustian, K. (2002) *Global Biogeochemical Cycles* **16**.

[50] Ketcham, C. (2019) *This Land: How Cowboys, Capitalism, and Corruption are Ruining the American West.* (Viking, New York), p. 432.

[51] Copeland, S. M, Davies, K. W, Boyd, C. S, & Bates, J. D. (2021) *Ecospghere* **12**.

[52] Soussana, J. F, Tallec, T, & Blanfort, V. (2010) *Animal* **4**, 334–350.

[53] Stewart, C. E, Paustian, K, Conant, R. T, Plante, A. F, & Six, J. (2008) *Soil Biology and Biochemistry* **40**, 1741–1750.

[54] Briske, D. D, Bestelmeyer, B. T, Brown, J. R, Fuhlendorf, S. D, & Polley, H. W. (2013) *Rangelands* **35**, 72–74.

[55] Briske, D. D, Ash, A. J, Derner, J. D, & Huntsinger, L. (2014) *Agricultural Systems* **125**, 50–53.

[56] conant, R. T, Cerri, C. E. P, Osborne, B. B, & Paustian, K. (2017) *Ecological Applications* **27**, 662–668.

[57] Sanderman, J, Hengl, T, & Fiske, G. J. (2017) *Proceedings of the National Academy of Sciences* **114**, 9575–9580.

[58] Song, J, Wan, S, Peng, S, Piao, S, Ciais, P, Han, X, Zeng, D.-H, Cao, G, Wang, Q, Bai, W, & Liu, L. (2018) *Ecosphere* **9**.

[59] Rotich, H. K, Onwonga, R, Mbau, J. S, & Koech, O. K. (2018) *Journal of Rangeland Science* **8**, 143–154.

[60] Shrestha, G & D.Stahl, P. (2008) *Agriculture, Ecosystems & Environment* **125**, 173–181.

[61] Qasim, S, Gul, S, Shah, M. H, Hussain, F, Ahmad, S, Islam, M, Rehman, G, Yaqoob, M, & Shah, S. Q. (2017) *International Soil and Water Conservation Research* **5**, 62–68.

[62] Wade, C, Sonnier, G, & Boughton, E. H. (2022) *Rangeland Ecology & Management* **80**, 10–17.

[63] Yu, L, Sun, W, & Huang, Y. (2021) *Agriculture, Ecosystems & Environment* **320**, 107605.

[64] Sitters, J, Kimuyu, D. M, Young, T. P, Claeys, P, & Venterink, H. O. (2020) *Nature Sustainability* **3**, 360–366.

[65] Bai, Y & Cotrufo, M. F. (2022) *Science* **377**.

[66] Ratajczak, Z, Collins, S. L, Blair, J. M, Koerner, S. E, Louthan, A. M, Smith, M. D, Taylor, J. H, & Nippert, J. B. (2022) *Proceedings of the National Academy of Sciences* **119**.

[67] Pineiro, G, Paruelo, J. M, Jobbagy, E. G, Jackson, R. B, & Oesterheld, M. (2009) *Global Biogeochemical Cycles* **23**.

[68] Jr., E. R. H, Kelly, R. D, Smith, W. K, Fahnestock, J. T, Welker, J. M, , & Reiners, W. A. (2004) *Environmental Management* **33**, S432–S441.

[69] Holdo, R. M, Holt, R. D, Coughenour, M. B, & Ritchie, M. E. (2007) *Journal of Ecology* **95**, 115–128.

[70] Paruelo, J. M, Pineiro, G, Baldi, G, Baeza, S, Lezama, F, Altesor, A, & Oesterheld, M. (2010) *Rangeland Ecology & Management* **63**, 94–108.

[71] McSherry, M. E & Ritchie, M. E. (2013) *Global Change biology* **19**, 1347–1357.

[72] Carey, C. J, Weverka, J, DiGaudio, R, Gardali, T, & Porzig, E. L. (2020) *Geoderma Regional* **22**, e00304.

[73] Wang, T, Kreuter, U, Davis, C, & Cheye, S. (2024) *Proceedings of the National Academy of Sciences* **121**.

[74] of Agriculture, U. S. D. (2021) Quick stats (`https://quickstats.nass.usda.gov/`).
